# Effect of Various Recycled Construction and Demolition Fines on Cementitious Mortars Rheological and Mechanical Performance

**DOI:** 10.3390/ma19173726

**Published:** 2026-09-01

**Authors:** Joanna Julia Sokołowska, Bartłomiej Przybyszewski

**Affiliations:** 1 Department of Building Materials Engineering, Faculty of Civil Engineering, Warsaw University of Technology, 00-637 Warsaw, Poland; 2 Faculty of Materials Science and Engineering, Warsaw University of Technology, Wołoska 141, 02-507 Warsaw, Poland; bartlomiej.przybyszewski@pw.edu.pl

**Keywords:** recycled concrete fine, construction and demolition waste, blended cement, supplementary cementitious materials, low-carbon materials, sustainability, circular construction, colorimetry

## Abstract

**Highlights:**

- CDW fines from various ceramic units, AAC units and mixed mineral wastes were investigated as SCM candidates.
- Mortar properties depended on PSD and the pozzolanicity of applied recycled fines.
- Recycled red clay ceramic fines delivered the best mechanical performance.
- Colorimetry can be considered as a preliminary measure for assessing the quality of dust from recycled ceramics.

**Abstract:**

This study presents an extensive investigation on the rheological and mechanical performance of cementitious mortars incorporating fines derived from recycled construction and demolition waste (CDW), including masonry units (bricks and ceiling blocks), autoclaved aerated concrete (AAC) units, and mixed demolition residues obtained during the renovation of a pre-war tenement house located in Warsaw, Poland. The CDW fines were assessed for their potential as secondary cementitious materials, SCMs. The proposed material concept aimed to integrate CDW into sustainable binder systems and reduce Portland clinker consumption, thereby lowering CO_2_ emissions of the concrete industry. Binder blends were produced by replacing 25 wt%. of Portland cement with recycled fines, achieving a 33–36% fines/clinker mass ratio. Recycled fines obtained by grinding CDW materials were characterized in terms of morphology, specific gravity, PSD, specific surface area, pozzolanic activity, pH and—in the case of recycled AAC—also carbonation. Mortars mixes were tested for consistency directly after mix preparation, while hardened composites were tested for density, compressive strength and flexural strength after 28 and 90 days of water curing. Statistical analysis showed that the origin of recycled fines affected mortar performance, with pozzolanic activity identified as the key factor. However, all analyzed composites were characterized with good mechanical strength. Overall, the performed investigation confirmed the suitability of analyzed CDW fines for clinker-reduced, low-carbon binders and highlighted their potential (especially in the case of ceramic fines) to support circularity in construction.

## 1. Introduction and Research Significance

The construction sector is currently struggling with environmental challenges including substantial CO_2_ emissions from cement manufacturing and the increasing volume of construction and demolition waste (CDW). Annual cement production is currently estimated at 4.1 billion metric tons [1] and is said to be responsible for 6–8% of the total world’s CO_2_ emissions [2]. Simultaneously, CDW generation, including primarily concrete, ceramics and glass waste generation, is estimated at over 2 billion metric tons [3,4,5,6,7,8,9], with a significant portion of waste improperly processed or directed to landfills without any further processing [8,9]. At the same time, CDW generated in EU countries is estimated to be nearly 40% of the total waste [10,11]. These high numbers demonstrate the enormity of CO_2_ emissions and CDW problems that need to be solved, which have been already highlighted in the Circular Economy Action Plan (CEAP) [12] as the crucial issues to be solved.

A solution to both challenges may be the re-use of mineral construction and demolition wastes in concrete production. Currently, the main method of implementing mineral CDW into concrete technology is the use of fragmented wastes (e.g., crushed concrete or ceramics) to produce recycled, primarily coarse (>4 mm) aggregate [13,14,15,16,17,18]. This approach is not free from problematic issues, which include the high porosity and water absorption of such recycled aggregates, as well as lower interfacial adhesion between the aggregate and the cement paste in newly manufactured concrete [13,18]. Meanwhile, recently, there has been a growing interest in another approach to the use of mineral CDW, i.e., searching for waste materials with residual pozzolanic activity and using them as mineral additives for concrete, or as constituents of new types of cement, or as supplementary cementitious materials, SCMs [7,19,20,21,22,23,24,25,26,27,28,29,30,31,32,33,34,35,36,37,38,39,40,41,42,43,44,45,46,47].

The use of recycled concrete fines [7,19,20,21,22,23] or recycled ceramic and silicate fines [7,24,25,26,27,28,29,30,31,32,33,34,35,36,37,38,39,40,41,42,43,44,45,46,47] as SCMs represents a growing trend in sustainable concrete production. The incorporation of such recycled fines can be done to partially replace clinker, thus reducing CO_2_ emissions, and enhance the circularity of the construction sector [23]. Prior research studies [7,19,20,21,22,23,24,25,26,27,28,29,30,31,32,33,34,35,36,37,38,39,40,41,42,43,44,45,46,47] (discussed in Section 2) have demonstrated that blends of Portland cement with such fines can be a promising alternative to ordinary Portland cements, OPC, or even composite cements incorporating industrial by-products such as siliceous and calcareous fly ashes or ground granulated blast furnace slag, that are now used in cement and concrete technology [48]. Especially, the availability of fly ashes (the by-products of hard coal and lignite combustion in power plants or cogeneration plants) is steadily diminishing due to the global transition away from coal-fired energy production in favor of low-emission alternatives such as nuclear and renewable sources [49].

Thus, incorporating recycled CDW fines into cementitious composites formulations as partial substitutes for Portland clinker facilitates the integration of these wastes into sustainable materials, which is a relatively new way of utilizing them. At the same time, using recycled fines can lead to a significant reduction in the carbon and environmental footprints of concrete and concrete-based structures [50]. The potential of fines derived from CDW such as clay masonry units (i.e., bricks and tiles), calcium silicate masonry units, or aerated concrete units is less explored than in the case of recycled concrete fines—both in terms of using them as the F constituent of Portland composite cements (CEM II) and composite cements (CEM IV) discussed in the new European standard EN 197-6 [51], and in terms of using them as SCMs. This is partly because this new cement’s constituent F stands for a recycled fine derived from recycled aggregates that, according to standard EN 12620 [52], should not contain remains of clay masonry units and/or calcium silicate masonry units and/or aerated concrete units in the amount exceeding 10% by mass. Thus, the validation of such recycled CDW materials’ applicability as potential SCMs seems to be a more reasonable approach and requires further investigation.

The authors addressed this research gap through a program based on waste materials collected during a partial renovation of a post-war-built tenement house located in Warsaw, Poland. The material was collected mainly from the ca. 60-year-old multi-ribbed ceiling using Ackerman technology. Moreover, the tenement house was renovated in previous decades and supplemented with newer repair materials in places of local damage. Therefore, the collected material is a mixture of various old construction products used at different times, including original elements of the Ackerman technology ceiling (specifically: Ackerman ceramic ceiling blocks, concrete/mortar overlay and cement–lime ceiling plaster) and other materials, such as different types of solid bricks (produced at different times) or pre-cast elements made of lightweight autoclaved aerated concrete, AAC.

The waste was delivered from the renovation site in the form of the mix of the crushed fragments of the above-mentioned materials. Part of the waste was then separated into materials: two types of ceramic bricks, Ackerman’s ceramic blocks, autoclaved aerated concrete blocks and cement–lime mortar, while the other part was left as delivered (and later referred to as the “mixed aggregate”). All materials were then fractioned using sieves conforming to standard EN 933-1 [53]. The fine wastes were obtained through grinding. Then the fines were assessed for their viability as a potential SCM in mineral cementitious blends prepared by substituting 25 wt% of Portland cement CEM I 42.5R (Cementownia “Odra” S.A., Opole, Poland) with recycled fines, resulting in a 33–36% fines-to-Portland clinker mass ratio.

Considering the findings reported in the scientific literature on cementitious mortars incorporating CDW fines (discussed in detail in the Section 2), most studies apply mass-based replacement of Portland cement with recycled fines at arbitrarily selected levels of 10%, 20%, and 30%. In the present work, a different approach was adopted. The substitution level was related to the actual proportions of cement constituents that exhibit pozzolanic activity and can serve as alternatives to Portland clinker. This strategy is particularly justified in light of the EN 197-6 European standard, which classifies recycled concrete fines (F)—potentially containing minor amounts of recycled ceramic and AAC fines as recognized constituents of Portland composite cements (CEM II) and composite cements (CEM IV). Accordingly, the replacement level of Portland cement with recycled CDW fines was set at 25%, which corresponds to a clinker substitution rate of 24–25%, i.e., a level within the 21–35% range specified for the F-type constituent in CEM II/B-F cements. Adopting this framework also enabled the evaluation of the pozzolanic reactivity of the recycled CDW fines used in this study.

The following sections contain details on the material characterization of the recycled CDW fines and their comparison to the analogous characteristics of the Portland cement they were intended to replace. The results of tests on fresh and hardened mortars with such blended binders are discussed in the context of the findings described in the scientific literature, as well as in the context of expectations regarding the characteristics of low-carbon composites implemented within the framework of circular construction policy.

## 2. Recycled CDW Fines and Their Potential for Use in Cementitious Composites

One of the reasons for using recycled construction and demolition wastes fines, including masonry ceramic fines and aerated concrete fines, as the materials supplementing the Portland clinker is that these fines may exhibit favorable compositions that are chemically compatible with Portland cement systems, rich in silica (preferably amorphous), calcium or alumina phases. Han et al. [7] have already reported that the recycled concrete filler they used as a SCM in cement pastes and mortars contained significant amounts of carbonates (calcite and dolomite), which supported the development of stable carbonate hydrates and helped the paste’s microstructure become denser over time. When the above-mentioned filler was combined with ceramic waste, it gained more gypsum and small quantities of reactive aluminosilicates, such as kaolinite, which affected the early hydration processes and contributed to microstructural improvement [7]. Thus, the authors investigated whether similar benefits could be achieved using the recycled fines examined in the present study.

Analysis of the scientific literature published in recent years generally confirmed that blends of Portland cement with recycled CDW fines can be promising alternatives to ordinary Portland cements, although many of the studies involved composites containing small amounts of fines, usually 10–30% of the total binder mass [7,24,25,26,27,28,29,30,31,32,33,34,35,36,37,38,39,40,41,42,43,44,45,46,47]. The following sections contain a synthetic analysis of the effect of different dosages of recycled masonry ceramics fines and autoclaved aerated concrete fines (i.e., fines of origin analogous to those used in the authors’ own research presented in this paper) on selected properties of cementitious composites.

### 2.1. Recycled Brick Fines in Cementitious Mortars and Concrete

Previous studies indicate that recycled brick fines can partially replace cement in mortar and concrete applications. Ceramic brick fines’ pozzolanic reactivity may be attributed to relatively high contents of reactive (amorphous) silica, alumina and iron oxide, often exceeding together 70% [7] or even 80 wt% [25], and may promote the formation of additional calcium silicates hydrate phases (C-S-H), monosulfate and ettringite (AFm/AFt), hydrogarnet phases or calcium ferrite hydrate phases (C-F-H), contributing to cementitious matrix densification and, as a consequence, to additional mechanical strength development [54,55].

However, Siline [24] indicated the importance of the particle size distribution of artificial pozzolanas obtained from clay materials and recommended using powders of at least the same fineness as Portland cement so that the chemical and physical effects occurring in new blended cementitious matrices could be sustained on a similar level or be improved compared to pure OPC matrices. Otherwise, the fluidity and rheology of the new mix could be affected, resulting in improper microstructure formation. Similar conclusions were drawn in the context of recycled brick fines by Wu et al. [25] and Li et al. [26], who, after testing standard mortars where cement was substituted with red brick fines by 10%, 20% and 30 wt%, concluded that the presence of brick fines negatively affected the fluidity of the mix: deterioration was consistent with the increase in brick fine content, and the effect was stronger for finer powders.

**Figure 1:**
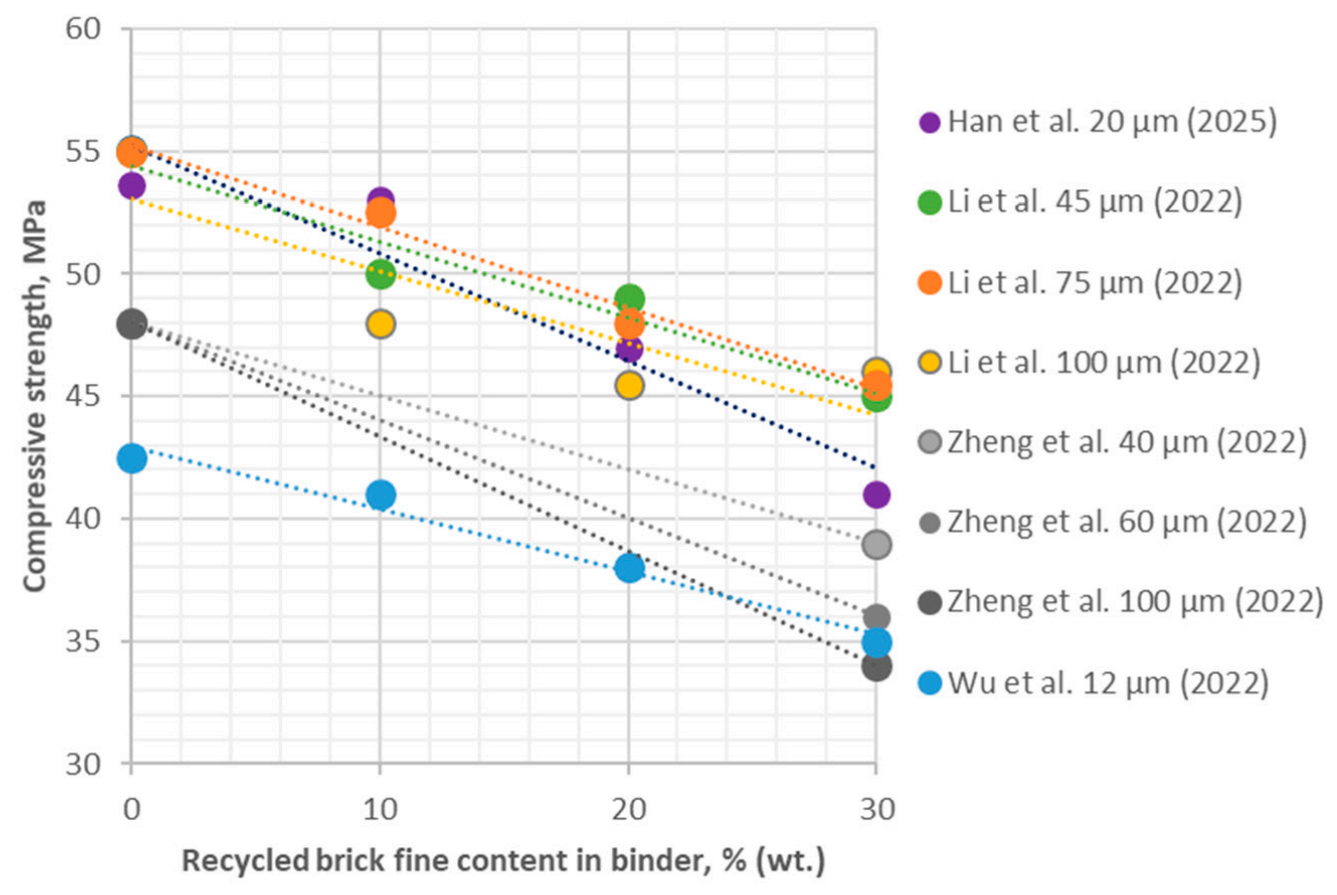
Compressive strength of 28-day-old mortars with binders consisting of Portland cement and recycled brick fines of various PSD (represented by median value in µm) mixed in cement/fines with mass ratios equal to 100:0, 90:10, 80:20 or 70:30 (elaborated on the basis of [7,25,26,27]).

A similar tendency with increasing brick fine content was observed in the case of the compressive strength with a maximum decrease of approx. 18% (17.6% in the case of Wu’s experiment and 18.2% in the case of Li’s experiment), while no deterioration was noted or the effect was practically negligible in the case of flexural strength. A more precise analysis of several research works [26,27,28,29] done to determine the effect of granulation of waste brick powders on the compressive strength of mortars showed that the finer the brick powder, the higher the strength of the mortar containing it, and the smaller the effect of strength deterioration with increasing brick powder content in binder mix. The greatest reduction in mortar compressive strength (29.2% lower compared to pure cement mortar’s strength) was obtained by Zheng et al. [27] in the case of 30% mass substitution of cement with brick fines of the coarsest grain size (median of 100 µm). Meanwhile, the same mass substitution with the finest grain size brick fines (median of 40 µm) resulted in an 18.8% decrease in compressive strength [25] (which stands in line with previously cited data [25,26]). Han et al. [7] also obtained a higher reduction in strength, i.e., 23.5%, in the case of 30% mass substitution of cement with brick fines; however, it is worth noting that they substituted quite fine Portland cement CEM I 52.5R of mode size of 10 µm with brick fines of mode value twice as high (20 µm), thus, the results are consistent with generally observed trends. Figure 1 presents the effects of OPC substitution with waste brick fines (of various grading) on the compressive strength of mortars measured after 28 days of curing. Considering the results for the reference standard mortars (conforming to standard EN 196-1 [56]), a clear distinction can be made between mortars with cements of compressive strength class 42.5 and 52.5.

The brick-derived fines used as SCMs were also analyzed in the context of their effect on concrete mix consistency. The study described in [30] showed that 5–10 wt% replacement of cement with wastes improved consistency (determined through the slump test). However, the PSD of those fines was not provided (it is only known that 91% of the brick fines passed through the 75 µm sieve, but no information about the mode or median was given), and taking into account that the amount of the recycled material was relatively small, it was hard to draw a clear conclusion about the positive effect of the brick fines on fresh mortar or concrete mixes.

The above-mentioned study contains also the results of destructive and non-destructive testing of hardened concretes, with brick fines showing slightly higher values of the compressive strength (higher by up to 2.7 MPa), split tensile strength (higher by up to 0.6 MPa), and flexural strength (higher by up to 0.75 MPa) of concretes with the fines compared to the pure cement ones. The authors of [30] explained that as a matrix densification by increasing C–S–H and stable ettringite formation. Nonetheless, given the low extent of binder modification and the lack of granulometric characterization of both brick fines and cement, it is difficult to relate these findings to the previously discussed results. Especially relevant because of the much lower specific gravity of brick fines compared to the applied cement specific gravity (of 2170 kg/m^3^ and 3150 kg/m^3^, respectively) in the case of mass substitution, the observed “densification” (demonstrated by the higher value of pulse velocity determined during non-destructive (NDT) ultrasonic testing [31]) effect could have resulted from the simple physical filling of voids in the mineral matrix with brick particles of larger size.

The above findings clearly indicate that in addition to determining the chemical composition (important, as it determines the products of potential reactions occurring between brick fines and the products of the hydration and hydrolysis of cement clinker phases), it is also necessary to examine the PSD of cement and fines that are to be their substitute before blending them, as the grading of both co-binders has a very large impact on the final composite’s technical properties.

### 2.2. Recycled Brick and Ceramic Fines in Other Cementitious Composites

The analysis of the state of knowledge also showed that attempts were made to mix small amounts of brick fines with fines from other ceramic materials and use such blends as potential SCMs. Such an approach was used, for example, in the case of high-strength concrete (HPC) [32], where cement substitution was applied at the level of 15 wt%, the substitute including one part of brick fines and two parts of kaolinite-based ceramic fines, thus a material rich primarily in SiO_2_, Al_2_O_3_ and Fe_2_O_3_, [33]. Such a ceramic blend, due to the content of amorphous silica, promoted the formation of additional hydration products, and consequently, a denser microstructure of the mineral matrix. These findings seem promising, yet it was also experimentally proven that the use of higher levels of cement substitution with the fines blend resulted in deterioration in the properties of hardened concrete.

Other attempts to use waste ceramic brick fines or white ceramic fines in composites with mineral matrices include geopolymers, i.e., alkali-activated systems, where ground ceramic wastes proved to be effective supplementary precursors in ternary binders containing also siliceous fly ash and ground granulated blast furnace slag. Liang et al. [34] demonstrated that substituting up to 75 wt% of fly ash with red brick fine in a geopolymeric system enabled the acceleration of the polymerization reaction rate and the deepening of the reaction degree, resulting in higher amorphous-phase C-(A)-S-H gel content, and later, in the increase in gel pores and the reduction of capillary pores volume, leading to the increased compressive strength and better durability of the composite. Similar findings were obtained by Sharmin et al. [35], as they proved that incorporating red brick fine into fly ash-GGBFS geopolymer-based mortar resulted in the formation of larger amounts of C-A-S-H and N-A-S-H gels, thus producing denser and more compact geopolymer matrix.

The performance of recycled brick fine has also been investigated in cement–ceramic-fine-lime ternary binders or ceramic fines–lime binary systems, where pozzolanic activity was additionally accelerated through the addition of hydrated lime, which also positively influenced the plasticity of the fresh mix. Historically, brick dust and crushed brick fragments have been incorporated into lime mortars to increase pozzolanicity and mitigate thermal degradation. Pachta et al. [36] proved through modern experimental methods that mortars containing brick residues retained long-term strength, exhibited reduced capillary absorption, and maintained structural integrity after exposure to elevated temperatures, making them suitable for restoration and fire-resistant construction applications. Shah et al. [37] reported that substituting cement with brick fine up to 10 wt% in such a self-compacting ternary system caused an improvement of mechanical strength at later ages, while the irregular, porous morphology of brick micro-particles caused a reduction in early-age shrinkage and enhanced microstructural compactness. Higher replacement ratios, however, necessitate careful optimization to avoid strength reduction.

The utilization of ceramic waste powders derived from ceramic blocks and ceramic tiles [38,39,40] in concrete has been also examined. Fines generated during the final polishing process of ceramic tiles contain more than 85% of SiO_2_ and Al_2_O_3_ and exhibit particle morphology like the one of Portland cement, making it suitable as a partial (up to 40 wt%) cement substitute [39]. The above-mentioned research studies have proved positive effects of such fines’ presence on the workability in the case of composites with low and medium strength. However, the inclusion of fines affected negatively the early strength and generally reduced the intensity of strength development, although—similarly to fly ash or ground granulated blast-furnace slag [48]—it provided satisfactory strength after 90 days, as well as reduced drying shrinkage strain after 120 days. Moreover, for concretes with recycled ceramic tile fines, improved resistance to chloride ion penetration, sulphates and carbonation, as well as increased electrical resistivity and decreased permeability (reflecting improved durability), were noted [38,39]. A performance index approach was then proposed to optimize the substitution of cement with ceramic tile fines, indicating 10–20 wt% replacement optimal in terms of workability and strength criteria, while 30–40 wt% replacement is optimal in terms of combined durability, workability, and strength criteria [39]. Additionally, it was stated that when combining recycled ceramic tiles’ dust fractions with recycled tiles fines and coarse aggregates, their contents should not exceed 20%, 60% and 30 wt% respectively of analogous traditional fillers. It was explained by much higher porosity, thus water absorption, of such ceramic recycled aggregates compared to natural aggregates, which significantly deteriorated the workability of the newly prepared concrete mixes [40].

### 2.3. Autoclaved Aerated Concrete Fines in Cementitious Composites

Autoclaved aerated concrete, widely utilized in masonry blocks and pre-cast elements due to its low density, high thermal insulation capability, and high fire resistance, generates substantial post-demolition waste that creates disposal and environmental challenges [41,42,43,44,45,46,47]. Consequently, increasing research attention has been directed toward recycling AAC residues, particularly in the form of finely ground recycled concrete fines [43,44,45,46,47]. Volk et al. [43] assessed the AAC as the so-called “open loop” material, as the recycled AAC fines can be easily incorporated into new AAC products. Radina et al. [44] reported after Lam [45] that AAC waste can replace up to 25% of natural sand in new AAC fabrication. Sun et al. [46] reported that increasing AAC recycled fines’ content notably affects rheology, foaming behavior, density, and strength, with high fineness enhancing gas-foaming efficiency, promoting the development of amorphous C–S–H and tobermorite (well-crystalline C-S-H) phases during hydrothermal curing, thus densifying the matrix that additionally strengthens the composite. Wang et al. [47] confirmed that the intrinsic porosity and microstructural complexity of AAC fines strongly influence mechanical and durability performance in recycled AAC-based materials. However, they indicated that particle fineness, binder selection, and replacement levels must be carefully optimized to develop high-performance and environmentally sustainable new composites with recycled autoclaved aerated concrete fines.

Except for one case, all of the above-analyzed studies involved mass substitution of Portland cement with CDW fines at a maximum of 30%. Moreover, the level of cement substitution with recycled fines was arbitrarily assumed at 10%, 20%, and 30 wt% for mortars, and 5–10 wt% for concretes. The authors decided to approach the substitution level differently, i.e., to refer to the actual shares of cement constituents with pozzolanic properties that are alternative to Portland clinker, especially since the new EN 197-6 standard recognizes recycled concrete fines (with some content of recycled ceramic fines, etc.) as full-fledged constituents of Portland composite cements CEM II and composite cements CEM IV. Therefore, the level of Portland cement substitution with recycled fines from CDW was assumed to be 25%, which corresponds to a substitution of Portland clinker of 33–36%, i.e., a level close to the content limit of the F constituent in cements CEM II/B-F. This approach also enabled the determination of the pozzolanicity levels of the analyzed recycled CDW fines.

## 3. Components Characterization and Mix Design

### 3.1. Qualitative and Quantitative Composition of Mortars with Recycled Fines

The subjects of the research were mortars with Portland cement partially substituted with recycled fines derived from CDW. The mortar constituents included Portland cement CEM I 42.5R, tap water, standard CEN sand and recycled fines obtained from ground CDW. The recycled fractions originated from various materials, namely:

- Ceramic red bricks,
- Ceramic yellow bricks (also referred to as “historic bricks”),
- Ceramic ceiling blocks used in multi-rib ceilings in Ackerman technology,
- Autoclaved aerated concrete (AAC) units,
- Mixed aggregate (containing the aforementioned materials and fragments of cement screed mortar and cement–lime plaster mortar).

All mortars were prepared according to the guidelines for standard mix design specified in EN 196-1, maintaining a water-to-binder ratio (*w*/*c*) of 0.50 and a binder-to-aggregate mass ratio (b/a) of 1/3. The substitution level of Portland cement with recycled aerated concrete fines was adopted on the basis of EN-197-6, specifying levels of mass substitution of Portland clinker with a new constituent “F” (i.e., recycled concrete fines) in new types of Portland composite cement. According to this standard, CEM II/A-F should contain 80–94% of Portland clinker and 6–20% of concrete recycled fines, while CEM II/B-F should contain 65–79% of Portland clinker and 21–35% of F fines. The authors assumed a 33–36% Portland clinker mass substitution with recycled autoclaved aerated concrete fines, thus a value close to the upper limit of the recommended range of F content. Such substitution was obtained by preparing a binder blend consisting of 75 wt% of Portland cement CEM I 42.5R (containing at least 95% of pure Portland clinker) [57] with 25 wt% of recycled aerated concrete fines. The adopted composition offered the additional benefit of testing the pozzolanic activity index of the AAC fines in accordance with the EN 450-1 European Standard [58]; thus, it allowed for optimizing the scope of experimental testing while simultaneously maximizing results for further analysis.

Given the optimized composition of the mortar with recycled AAC fines, the authors decided to repeat that quantitative composition also in the case of blends with the other analyzed recycled fines. This enabled a comparative analysis of all tested composites in terms of one factor, namely, the type of mineral fine. The compositions of all tested mortars (including the pure cement reference mortar) are summarized in Table 1.

### 3.2. Technical Characteristics of Standard Components of Tested Mortars

To isolate the effect of recycled fines on the test results, the comparative standard mortar was selected as the reference (REF) composite. The standard mortar was made from high-early-strength Portland cement CEM I 42.5R (Cementownia “Odra” S.A., Opole, Poland) conforming to EN 197-1 [57], CEN standard sand (KWARCMIX, Tomaszów Mazowiecki, Poland) conforming to EN 196-1 [56] and tap water conforming to EN 1008 [59]. The mixing water’s pH measured using a 781 pH/Ion Meter (Metrohm AG, Herisau, Switzerland) was 7.09. The applied CEN standard sand was a natural quartz sand with silica (SiO_2_) content of at least 98 wt%, rounded grains and a size distribution recommended as in Table 2. The characteristics of the Portland cement CEM I 42.5R applied according to the manufacturer’s specification are given in Table 3 and Table 4, which summarizes the statistical parameters, describing the distribution and the specific surface area of cement—all determined using a laser diffraction PSD analyzer, Horiba LA-300 (Horiba, Ltd., Kyoto, Japan). These data are discussed in detail and compared with the PSD elaborated for recycled CDW fines in the next section.

## 4. CDW Fines Additional Characterization

### 4.1. Recycled Fines Source Materials Characterization

The fines used in the study were collected during a partial renovation of a pre-war-built tenement house located in Warsaw, Poland. The material was collected mainly from the ca. 60-year-old multi-ribbed ceiling using Ackerman technology. Moreover, the tenement house was renovated in previous decades and supplemented with newer materials in areas of local damage. Therefore, the collected material was a mix of various construction and repair products used at different times, including original elements of the Ackerman technology ceiling (specifically, Ackerman ceramic ceiling blocks, concrete/mortar overlay and cement–lime ceiling plaster) and other materials, such as various types of solid bricks (produced at different times, including also re-used historic yellow bricks) or pre-cast elements of autoclaved aerated lightweight concrete. The above-mentioned materials were partially separated based on their quantitative contribution to the mix. Dominant materials were extracted and then tested as separate source materials of the recycled fines: common red brick, historic yellow brick, ceramic ceiling blocks, AAC cellular concrete, and cement–lime plaster. Physical characteristics were tested for these materials.

The characterization was done according to the procedures described in the EN 1936 standard [60], including: specific gravity (determined using the Le Chatelier volumenometer, Lab-Szkło s.c., Cracow, Poland), bulk (apparent) density, water absorption (determined by weight and by volume), porosity and water tightness (determined during water absorption test). Laboratory test results are given in Table 5 and Table 6—specific gravity values are the mean of three results; in the case of the remaining characteristics, the presented values are the means of six results. The data allowed a preliminary assessment of the quality of these CDW materials in the context of using them as parent materials to produce SCMs.

### 4.2. Ceramics Additional Characterization

The color of clay ceramics results from the chemical and mineralogical composition of the raw material and the transformations of iron-bearing phases during firing. Iron is the principal chromophore, and its optical effect depends strongly on the oxidation state, mineral host phases, and firing conditions. Under oxidizing conditions, iron is predominantly stabilized as Fe(III), promoting the formation of finely dispersed hematite (α-Fe_2_O_3_) responsible for the characteristic red to red-brown color of many ceramic building materials [61,62], like in the case of red ceramic bricks and red ceramic ceiling blocks analyzed in the presented study. Lower content of hematite or its poorer dispersion results in lighter, brownish or yellowish tones. Calcium carbonate content also exerts a strong influence on brick color development, as decarbonation of CaCO_3_ during firing is followed by the formation of Ca-rich silicate and aluminosilicate phases such as gehlenite, anorthite, wollastonite, and pyroxenes. These phases can incorporate iron into their crystal lattices or reduce the fraction of free hematite, thereby diminishing its coloring efficiency. As a result, calcareous bricks commonly exhibit buff, beige, or yellowish colors [61,63]—similar to the shade of the historic yellow bricks, analyzed in the presented study. Moreover, yellow coloration may be a result of limited oxygen availability, rapid heating, or dense microstructures during firing that hinder complete iron oxidation, allowing Fe(II) to persist locally and suppress hematite crystallization [61,62,63,64]. Thus, the colors reflect the stability and transformation of iron-bearing phases [64].

From an applied perspective, brick color is particularly important in the study of historic and archaeological masonry, as color variations often correlate with differences in manufacturing technology and material properties. Field-based investigations of historic masonry by Dizhur et al. [65] confirmed that historic yellow bricks typically exhibit higher total and open porosity, lower bulk density, and reduced compressive strength when compared with red bricks. They also demonstrated that the color-based classification of vintage solid clay bricks correlates with statistically significant differences in stiffness and strength, with lighter-colored units tending to show lower elastic moduli and compressive strength. Moreover, yellow bricks often displayed greater variability in mechanical performance than common red bricks, even within the same structure, which can be explained by the fact that in historic masonry contexts, firing control was limited. In general, the use of color as a first-order tool in diagnostics and material characterization can be very useful, since ceramic color variability is attributed to differences in firing intensity, pore structure, and mineral bonding rather than to color alone, although color remains a readily observable proxy for these underlying material features. The approach proved to be particularly useful where destructive sampling is limited or not permitted.

**Figure 2:**
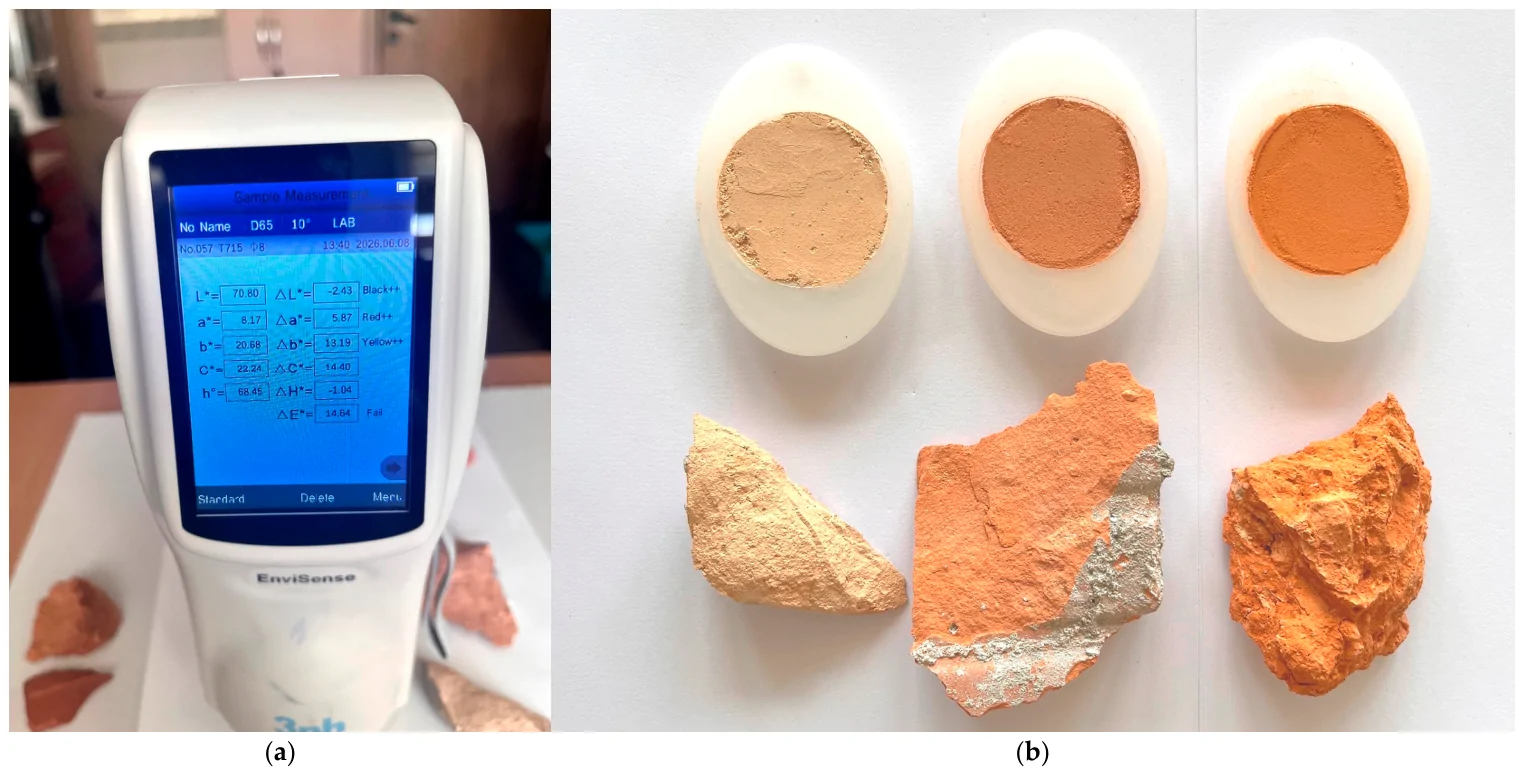
The device and recycled ceramics samples used in the coloring test: (**a**) 3nh NS800 spectrophotometer; (**b**) powdered and crushed samples of ceramics—from the left: “historic” yellow brick, Ackerman ceiling block and red brick.

A similar approach can be applied in the case of analyzing ceramic wastes properties. The ceramic CDW used in the presented study were tested using a 3nh NS800 spectrophotometer (Guangdong Threenh Technology Co., Ltd., Guangzhou, China)—Figure 2a.

The tests were done on the flat surfaces of the recycled ceramics fragments, as well as on the ready-to-be-applied ground fines that were compacted and leveled in the flat cylindrical mold to obtain a stable and smooth surface of the test sample—Figure 2b.

**Figure 3:**
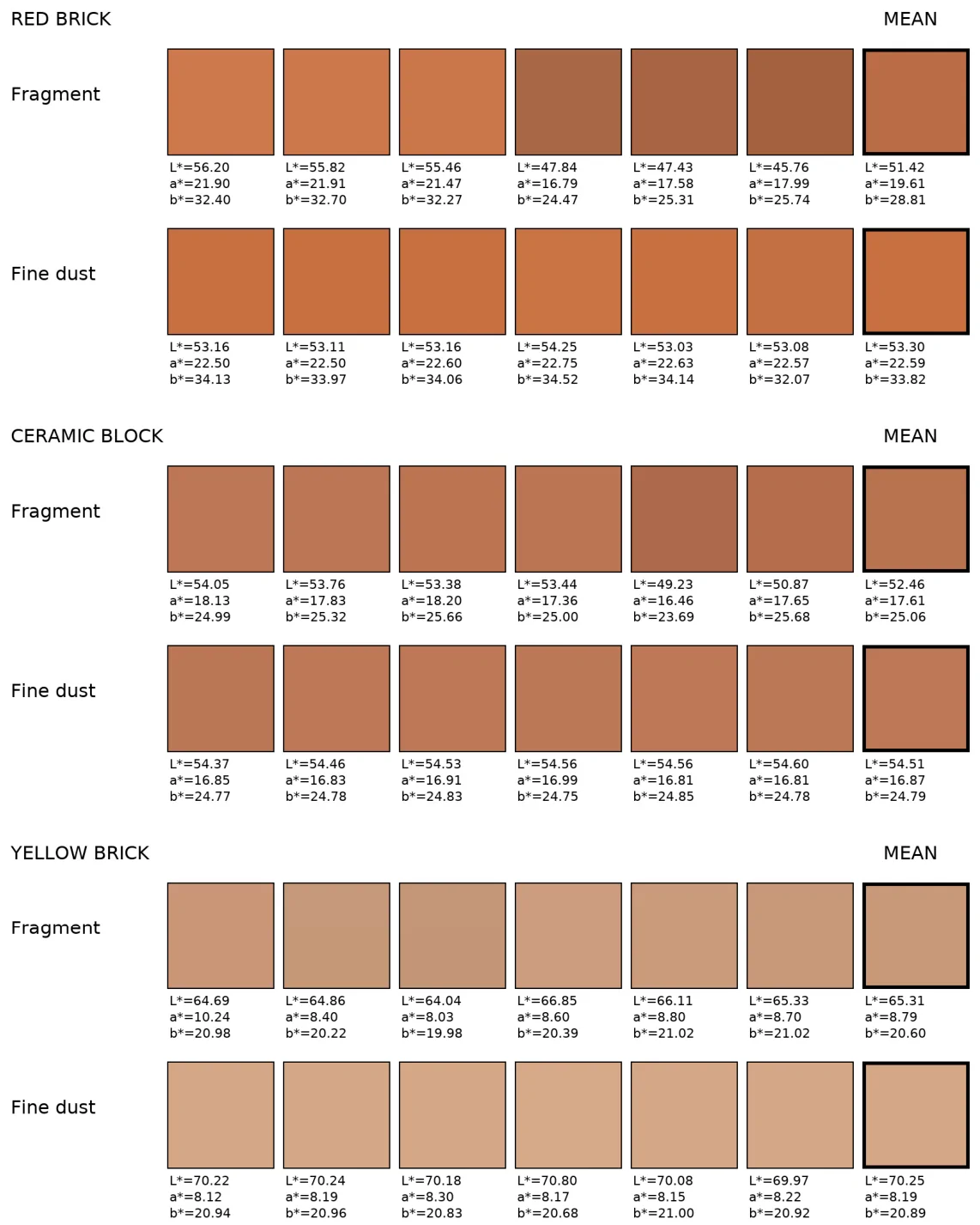
Graphical representation of 6 colors determined in subsequent measurements within the test of each recycled ceramic material and representation of the color corresponding to the mean L, a, b values; two series of tests show the effect of grinding on the color uniformity.

Spectrophotometric testing (illuminant and reference angle for input and output values were selected as D50 2°) allowed for color description based on three coordinate values (L, a and b) locating points in the colorimetric CIELAB space [66,67]. The L component corresponds to lightness, the a component corresponds to red and green, and the b component corresponds to blue and yellow. Each time, six measurements were taken at random locations, and ∆*E*^∗^_*ab*_ was calculated to compare the differences between the reference point and subsequent five test points to assess the homogeneity of the color according to the following formula:

(1) $${{\Delta E}^{*}}_{ab}=\sqrt{{\left({\Delta L}^{*}\right)}^{2}+{\left({\Delta a}^{*}\right)}^{2}+{\left({\Delta b}^{*}\right)}^{2}},$$

where ∆*L*^∗^, ∆*a*^∗^, ∆*b*^∗^ are differences in the values of coordinates of the two compared psychophysical points (tested and reference), and ∆*E*^∗^_*ab*_ is a color difference between the tested result and the reference result. L, a and b coordinates were then converted to the sRGB and CMYK shade scales using a “nix Color Sensor Converter” tool [66], enabling determination of the real colors. Figure 3 shows a graphical representation of all six colors determined in subsequent measurements within each test, as well as the color corresponding to the averaged L, a, b values. Table 7 presents statistical parameters (standard deviation, SD and coefficient of variation, CV) for the coordinates, mean values out of five results of ∆*E*^∗^_*ab*_ (considered a measure of the homogeneity of the color) and colors codes converted to the sRGB and CMYK scales determined for crushed fragments and ground fines.

The color difference parameter ∆*E*^∗^_*ab*_ can be interpreted as a measure of color homogeneity. According to the concept of the Just Noticeable Difference (JND) [68], color differences of ∆*E*^∗^_*ab*_ = 2.3 and higher correspond to the threshold at which variations become perceptible to an average observer, whereas lower values are generally considered visually negligible. The results obtained for ceramic fragments showed ∆*E*^∗^_*ab*_ values equal to 2.20 (thus visually negligible) for the yellow brick and ceramic block samples and 7.80 for the red brick samples, indicating noticeable color variability (compare Figure 3). In contrast, all ground ceramic fine powders exhibited ∆*E*^∗^_*ab*_ values below 1.0, namely, 0.74 for the yellow brick fine, 0.19 for the ceramic block fine and 0.27 for the red brick fine. This confirms that grinding substantially reduced color heterogeneity and produced visually uniform powders. From the perspective of SCMs, such homogenization is advantageous because it indicates a reduction in local differences associated with firing heterogeneity, weathering effects, pore distribution and mineral inclusions, resulting in powders of more reproducible characteristics.

The color observations together with the physical characteristics of the analyzed ceramics suggest that red bricks and ceiling blocks were produced from non-calcareous or weakly calcareous clays and were fired under predominantly oxidizing conditions, which favors the formation of hematite as the dominant iron-bearing phase. Such a mineralogical configuration is generally associated with relatively dense ceramic matrices, lower open porosity, and higher compressive strength, making red bricks mechanically robust and comparatively homogeneous. In contrast, historic yellow bricks were probably manufactured from calcareous clays, in which high CaCO_3_ contents modify phase assemblages and microstructural development during firing. The formation of calcium silicates and calcium aluminosilicates, combined with a reduced content of free hematite, should result in a more heterogeneous ceramic matrix.

Results obtained in the laboratory generally stand in line with the above. All tested ceramic materials (sourcing from modern and historic ceramic units) showed similar bulk density (in the range between 1546 and 1649 kg/m^3^), which was characterized by a similar coefficient of variation (6.8% on average). However, due to the differences in the specific gravity (between 2332 and 2734 kg/m^3^) which was a result of the different raw clay materials/firing conditions demonstrated by the ceramics’ colors [61,62,63,64,65], they presented various water absorption levels. In the case of red ceramics (brick and ceiling blocks), it was ca 0 wt% (and in the case of historic yellow ceramics, almost twice as much: 37.7 wt%. In terms of volumetric water absorption, the values were 32% on average for red ceramics and over 50% for yellow ceramics. Interestingly, both types of brick, modern red brick and historic yellow brick, showed similar levels of porosity and tightness: on average 35% and 0.65, respectively. The ceiling blocks showed higher volumetric porosity (43%) and lower water tightness (0.57). However, considering the use of this element in the structure, which does not have direct contact with water, these values were not surprising.

### 4.3. Cementitious CDW Additional Characterization

Meanwhile, autoclaved aerated concrete, AAC, due to its specific cellular structure, demonstrated significantly higher porosity (81.2%) and, consequently, higher water absorption (64.1 wt%) and lower tightness (0.19). As for specific gravity, the obtained value of 2478 kg/m^3^ is slightly higher than expected as—according to Neville [69]—the specific gravity of lightweight concrete takes values from the range between 2200–2400 kg/m^3^. With a bulk density of 464 kg/m^3^, the tested AAC corresponds to a typical low-density autoclaved aerated concrete, which is much lighter than the “LC” lightweight concrete with coarse aggregate described in [70], i.e., with a dry density from 800 kg/m^3^ to 2000 kg/m^3^.

The cement–lime plaster mortar specific gravity test showed a value of 2579 kg/m^3^, a result between the values typical for cement mortars and lime mortars (which according to Pavlík et al. [71] can achieve 2600–2800 kg/m^3^ after 90 days). In general, the obtained water absorption results of this mortar are also uncertain, as most of the plaster fragments had one side covered with several layers of different paints (including water impermeable emulsion), which must have influenced the test results. Considering this, and the fact that there were significantly fewer mortar fragments than other materials recovered from the construction site, the authors decided not to consider the cement–lime mortar as a separate parent material from which recycled fines would be obtained. A different approach was taken in the case of a starting mix of CDW material containing such mortar fragments. After grinding the entire mixed material, the specific gravity for such a mix was determined: it adopted a mean value of 2385 kg/m^3^ and was characterized by a standard deviation SD = 3 kg/m^3^ and a coefficient of variation CV = 0.1%. Such mixed fines were used as one of the potential SCMs.

**Figure 4:**
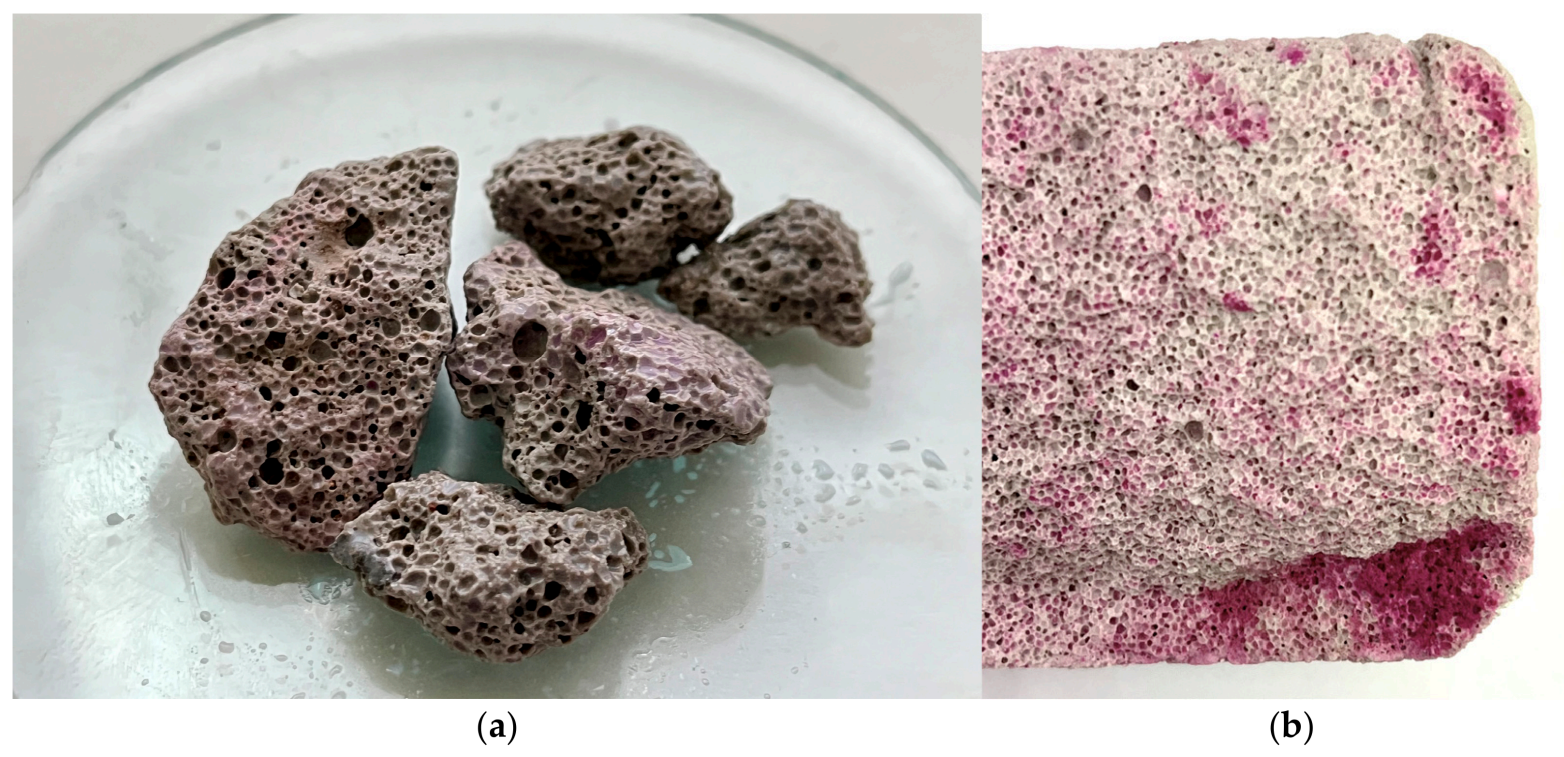
Fragments of the recycled AAC units treated with phenolphthalein indicator. The uncolored area indicates the carbonation of concrete with the pH lower than 8.3–8.5: (**a**) AAC, the parent material of the AAC fine under study—completely carbonated in its entire volume; (**b**) an example of younger, partially carbonated AAC tested by authors in the framework of other research.

In the case of recycled autoclaved aerated concrete, an additional test was performed to assess the level of its neutralization. The selected fragments of AAC units were treated with distilled water and phenolphthalein, an indicator of pH ~8.3–8.5, recommended for testing concrete carbonation by the standard EN 13295 [72]. The fragments were selected to represent the whole cross-section of the pre-cast units—i.e., from the interior and edges of the unit. As shown in Figure 4a, all tested fragments turned out to be colorless, thus completely carbonated. For comparison, the authors examined a fragment of another, younger AAC pre-cast element, which turned out to be partially carbonated—the magenta color indicates non-neutralized zones at the fracture of the specimen of this concrete (Figure 4b). This simple test demonstrated that the AAC utilized in the study must contain CaCO3 formed in the reaction of carbon dioxide with portlandite from the hardened paste [73,74]. This test was not suitable for determining the alkalinity of the other CDW materials, but the following section contains the results of the determination of pH of the aqueous solutions extracted from ground CDW fine powders.

### 4.4. Comparison of Physical and Chemical Properties of the Applied Recycled Fines

To prepare the recycled fines, the CDW rubble was fragmented and fractioned using a set of standard sieves because it was intended for use as fine and coarse recycled aggregate. The undesirable finest fractions, i.e., 0/0.125 mm and 0.125/0.25 mm, considered to increase the water demand ratio of the whole aggregate mix, were collected and subjected to grinding in a Fritsch PULVERISETTE 13 disk mill (Fritsch GmbH, Idar-Oberstein, Germany) with the assumption of obtaining powders of homogeneous grading and reproducible properties, in terms of maximal size, particle size distribution and specific surface area. The other expected effect of energy-intensive grinding was to mechanically activate the materials by exposing potentially active components (such as amorphous silica) present in larger fragments.

**Figure 5:**
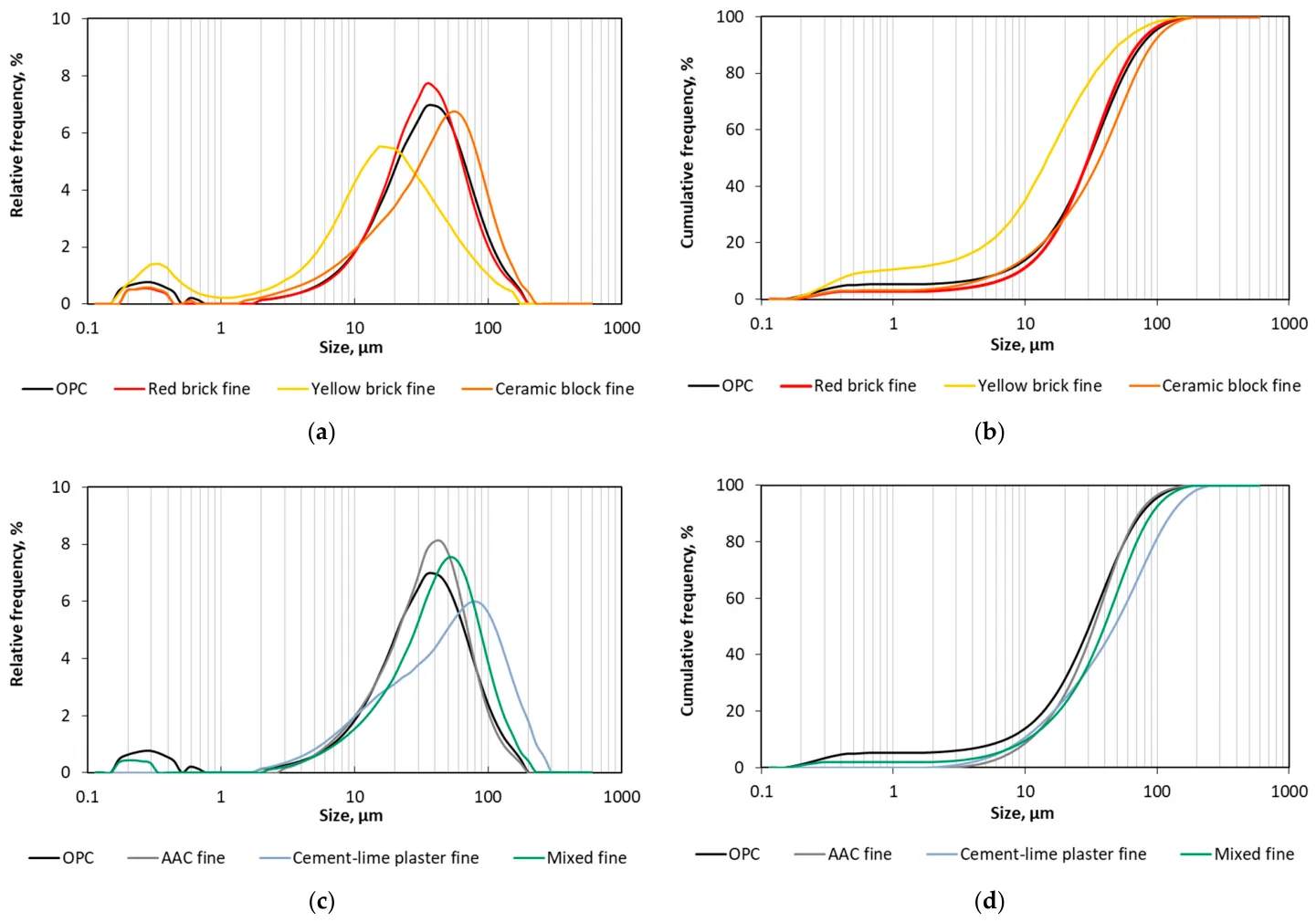
Particle size distribution of ordinary Portland cement (OPC) and ground recycled fines: (**a**) ceramic fines—relative frequency plot; (**b**) ceramic fines—cumulative frequency plot; (**c**) CDW fines containing cementitious phases—relative frequency plot; (**d**) CDW fines containing cementitious phases—cumulative frequency plot.

Table 8 summarizes the statistical parameters describing the PSDs and values of specific surface area (SPA) of the analyzed recycled fine materials obtained through grinding in the above-mentioned disc mill. As in the case of Portland cement, all distributions were determined on the basis of measurements performed in a laser diffraction particle size analyzer, Horiba LA-300 (Horiba, Ltd., Kyoto, Japan), using the laser diffraction phenomenon and Mie light scattering theory (LST) [56,75]. The device operated by passing laser beams through a 0.2% sodium hexametaphosphate (CAS: 1012-56-8) solution with particles of tested fine material dispersed by ultrasonics. The operating range of the device enabled measuring the particles size in micrometers, but SPA (calculated from the PSD, assuming the spherical shape of the particles [76,77]), due to the very high values, was expressed in cm^2^/cm^3^. The graphical representation of each PSD is given in one of the following figures. Figure 5a,b show a comparison of the grading curves of Portland cement and ceramic fines, while Figure 5c,d show a comparison of the grading curves of cement and CDW fines containing the hydrated cement phases—AAC fine, cement–lime plaster fine and mixed CDW fines.

By optimizing the grinding parameters, the authors managed to obtain fines with a grain size distribution similar to that of the Portland cement used in the study. This is particularly evident when comparing the median and mode values of their particle size distributions (see Table 4 and Table 8), with almost all distributions (except for the PSD of cement–lime plaster fines) being bimodal with a distinct presence of particles smaller than 1 µm (Figure 5). Considering the cement’s PSD, with a median of 30.5 µm and mode of 36.5 µm, the most similar in this respect are fines derived from recycled red brick (median: 30.7 µm, mode: 36.6 µm) and AAC units (median: 33.0 µm, mode: 36.8 µm), followed by fines from ceramic blocks (median: 36.7 µm, mode: 54.9 µm) and from mixed materials (median: 39.8 µm, mode: 46.4 µm). These four materials also had the most similar coefficients of variation of PSDs, with CV values ranging from 68.9 to 78.7. For comparison, the CV for the cement’s PSD was 78.7.

The finely ground powder from yellow clay ceramics, however, turned out to be significantly finer than cement, with a main mode clearly shifted to 14.2 µm and significantly lower D_10_ (0.7 µm) and D_90_ (51.3 µm) characteristics. Thus, this material is almost entirely made of particles considered silt-sized (<2 µm) or dust-like (2–63 µm) [78,79]. This may have two effects in the context of subsequent analysis: (1) negative, because particles smaller than 63 µm exhibit dominant surface-related phenomena, such as increased specific surface area (here, 1.7 times greater than that of cement—see SPA values in Table 4 and Table 8) and a higher tendency for agglomeration and airborne dispersion, or (2) positive, because mechanically activated particles may show enhanced reactivity. In contrast, the coarsest and most different from cement was the fine powder obtained from ground cement–lime plaster mortar, which also contained impurities in the form of paints used to coat the ceiling plaster layer.

**Figure 6:**
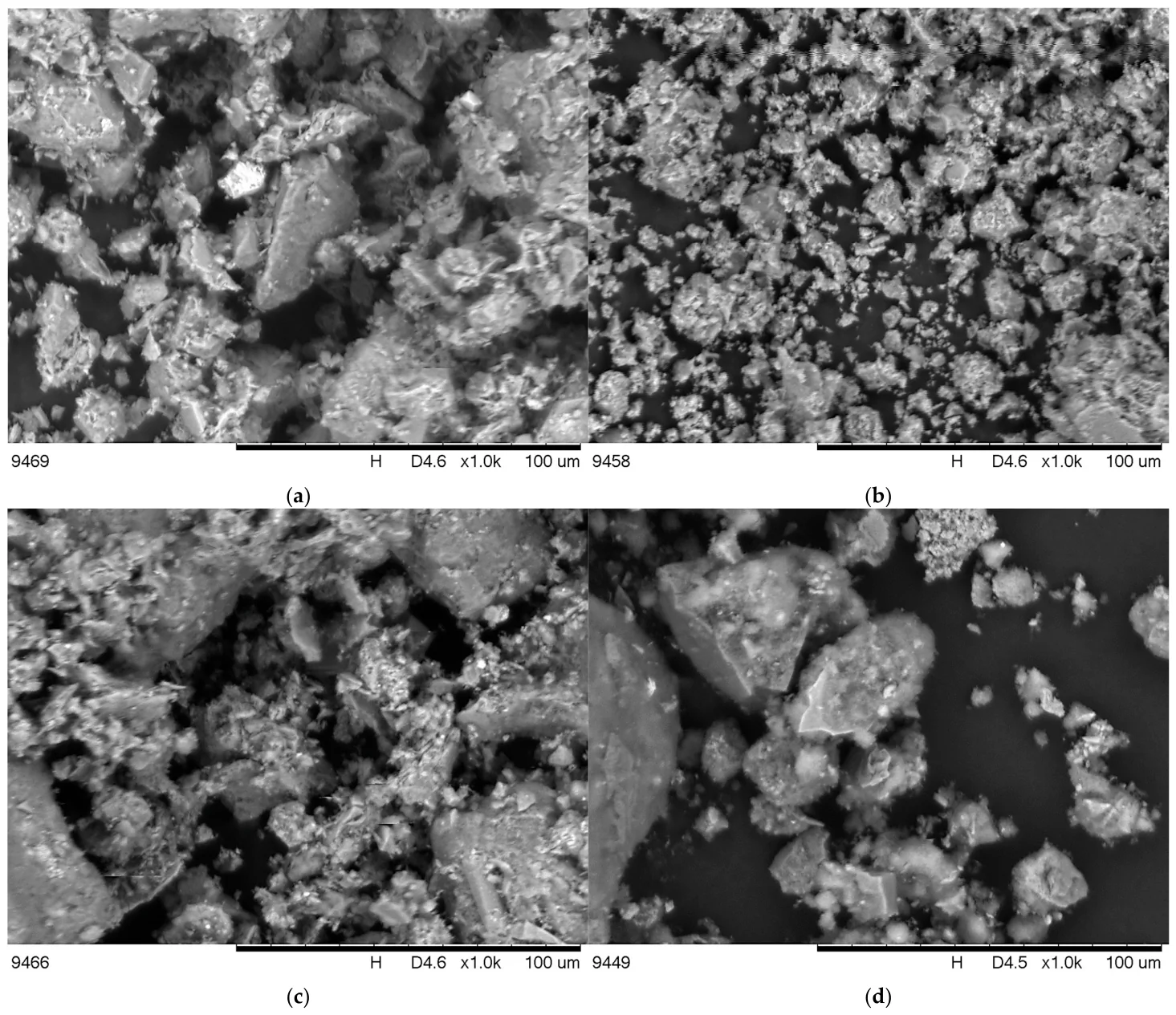

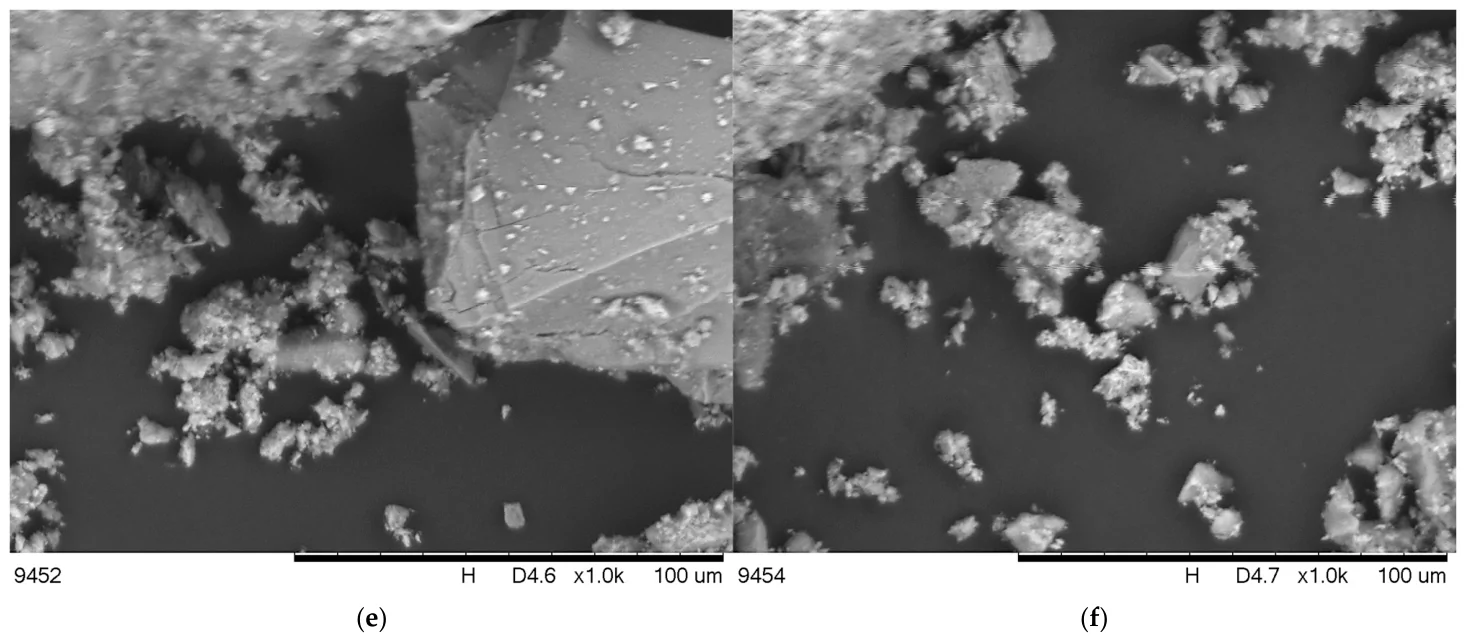
SEM micrographs (magnification: 1.0k×) of tested CDW fines: (**a**) red brick fines; (**b**) yellow “historic” brick fine; (**c**) ceramic block fine; (**d**) AAC units fine; (**e**) cement–lime plaster fine; (**f**) mixed CDW fine.

The results obtained using a laser diffraction particle size analyzer were confirmed by the microscopic observations. In Figure 6, one can see the morphology of the recycled fines. The micrographs were obtained using a Hitachi TM-3000 Scanning Electron Microscope (Tokyo, Japan) using a back-scattered electron (BSE) detector. Although the particle shape of all the fines appears similar, i.e., angular particles with sharp edges resulting from mechanical grinding in a disc mill, there are distinct differences in the particle size of yellow brick fine (Figure 6b) and cement–lime plaster (Figure 6e) fine compared to the other CDW fines. As revealed by the PSD analysis, yellow brick fine was by far the finest ground, while cement–lime plaster mortar the coarsest.

At this stage of the experiment, considering the physical parameters, granulometry, and morphology, but above all, the negligible amount of fines from the cement–lime plaster mortar compared to the total mass of CDW recovered from the renovation site, the authors decided not to treat recycled cement–lime plaster as a separate material tested in the context of a potential SCM. However, this waste was not eliminated, as it was still the component of the mixed CDW and fines derived from this mix.

To assess the potential CDW fines as SCMs, the first step was to measure their pH to determine their residual alkalinity, which could contribute to the overall alkalinity of a new cementitious blend with such materials, and then their pozzolanicity was assessed by determining activity index values.

**Figure 7:**
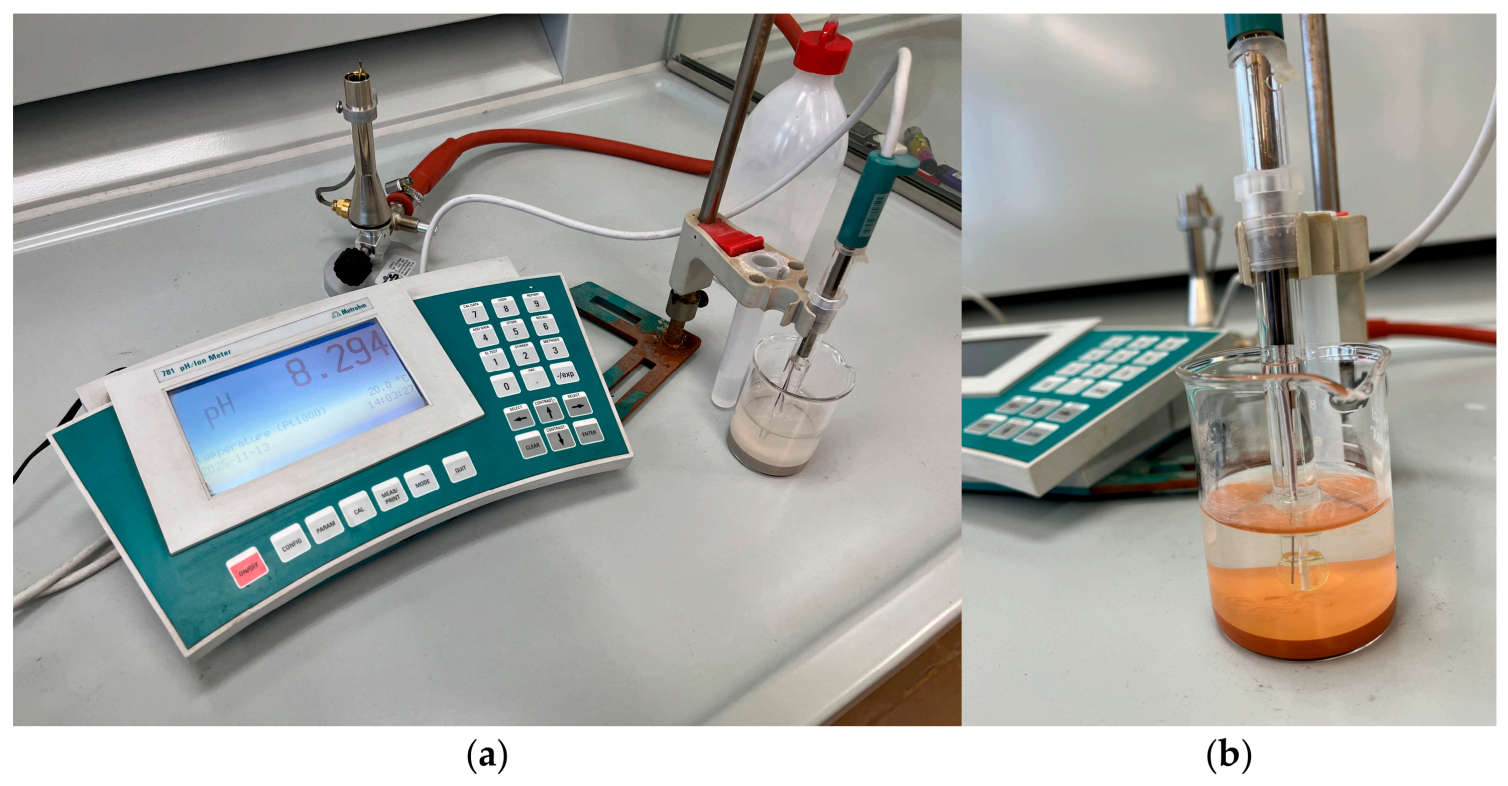
Determination of pH of aqueous solution washed out from CDW fines following the ISO 1039 procedure: (**a**) Metrohm 781 pH/Ion Meter equipped with a glass electrode and temperature sensor; (**b**) the electrode in the tested aqueous solution with ground red brick fine.

To measure the pH of the powdered materials, according to the procedure outlined in ISO 10390 [80] standard, it was necessary to prepare the aqueous extracts. These were 1:5 (vol.) CDW fines suspensions in distilled water mixed thoroughly and left to sediment to obtain a clear extract. The pH was measured using a 781 pH/Ion Meter (Metrohm AG, Herisau, Switzerland) equipped with a glass electrode and temperature sensor (Figure 7).

All ground CDW-based fines showed an alkaline reaction as expected; however, the pH took various values (Table 9). The lowest values were recorded for suspensions prepared from yellow-brick fines: pH = 8.24 (measured in temp. 18.1 °C), and in the case of the suspension of fines derived from AAC units: pH = 8.29 (measured in temp. 20.0 °C), which was not surprising. The yellow brick was the oldest of the tested materials, derived from the building that was constructed between 1936 and 1937 (and partially reconstructed around year 1950), and pre-war bricks typically exhibit reduced alkalinity due to long-term leaching of soluble alkalis and progressive carbonation during decades of service life. These processes lower the pH and stabilize the ceramic matrix by converting reactive alkaline phases into less alkaline carbonates [81]. This is generally a positive effect from the perspective of conservation work (such bricks show improved chemical compatibility with conservation-grade mortars; moreover, the reduced alkalinity also limits salt-related deterioration and surface efflorescence in reused masonry [82]), but not necessarily from the point of view of their potential use as SCM. Meanwhile, the result obtained for AAC confirmed the findings of the carbonation test, described in the previous section, where it was experimentally demonstrated that the AAC was completely neutralized in terms of carbonation, as the phenolphthalein indicated its pH as lower than 8.3–8.5. In contrast, for the suspensions of red brick fine, ceramic blocks fine and mixed fines, the registered pH adopted values from the range 9.1–10.1, confirming the residual alkalinity that could contribute—at least to some extent—to maintaining a high pH environment in the new mortars, which is essential for Portland cement hydration and the potential stability of the steel reinforcement [54,55,83,84,85].

**Figure 8:**
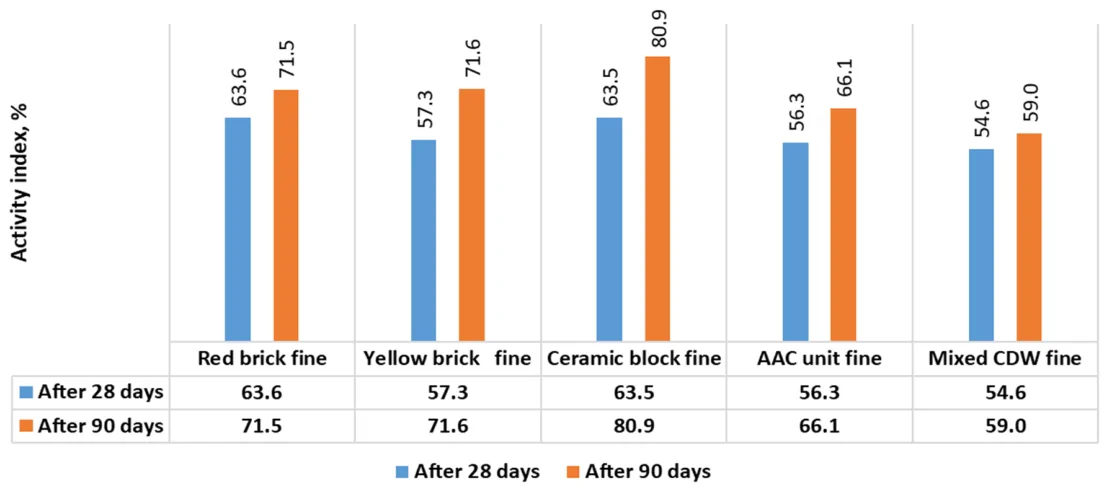
The activity index of tested CDW fines after 28 days and after 90 days of water-curing.

The pozzolanic activity, expressed by the activity index, of the analyzed CDW fines was tested according to the method described in the EN 450-1 European standard that assumes the preparation of standard mortars, in which Portland cement is replaced by the potentially pozzolanic tested material in an amount of 25 wt%. The test is performed on the sets of three prisms of size 40 mm × 40 mm × 160 mm, first broken in the three-point bending test and then subjected to compressive strength test. The compressive strength is determined after 28 days and after 90 days of water curing. The values obtained for composites with tested pozzolanic material are compared to analogous data obtained for pure cement reference mortars—expressed as the percentage of the reference mortar’s strength. The activity index values obtained for the analyzed CDW fines after 28 days and after 90 days of water-curing are shown in Figure 8.

According to the EN 450-1 criteria, the level of pozzolanic activity for a mineral material to be considered as a type II active concrete additive is attained when the 28-day activity index reaches at least 75% and the 90-day activity index reaches at least 85%. None of the tested fines reached these limits; thus, they cannot be classified as the type II additives. However, several of the tested materials showed quite high pozzolanic activity, which gives very good prospects for their use as SCM for concrete. The red clay ceramic fines showed the highest—and surprisingly identical—pozzolanic activity, with the 28-day activity index of 64% and 90-day activity index of 72–81%, which was quite close to the minimum values set for type II additives. This observation is consistent with the higher alkalinity measured for these materials, although pozzolanic activity is also affected by mineralogical composition and firing history. The results obtained for yellow brick fine were somewhat surprising. Despite its very fine grinding, the activity index value after 28 days was lower than for other clay ceramic materials. However, after 90 days, these values became evener. The fines that contained hardened cementitious phases showed lower values of 54–56% in the case of the 28-day activity index, and of 59–66% in the case of the 90-day activity index. However, these values can be still considered quite high, taking into consideration the origin and the age of the tested materials, and promising in the context of their potential as SCMs.

## 5. Cementitious Mortars with CDW Testing Methods

For all cementitious mortars, the set of technical properties was determined as follows: consistency (plasticity) of the fresh mortar, apparent density (of hardened mortar), flexural strength (tensile strength when bending), and uniaxial compressive strength. The properties of all mortars were determined after 28 days of curing (all specimens were demolded 24 h after casting and then kept in the water in laboratory conditions), while for mortars with selected recycled CDW fines, the additional testing was performed after 90 days of curing (additional two months of water-curing in laboratory conditions).

### 5.1. Consistency (Plasticity) of the Fresh Mix

The consistency of the fresh mortar mixes was determined using a flow table shaker. The test was performed directly after mixing, corresponding to the initial testing time for rheological measurements, following the method described in the EN 1015-3 European Standard [86]. The test result was the flow diameter, the mean of two diameters measured in perpendicular directions after shaking the fresh sample of mortar.

### 5.2. Density and Mechanical Strength of Mortars

**Figure 9:**
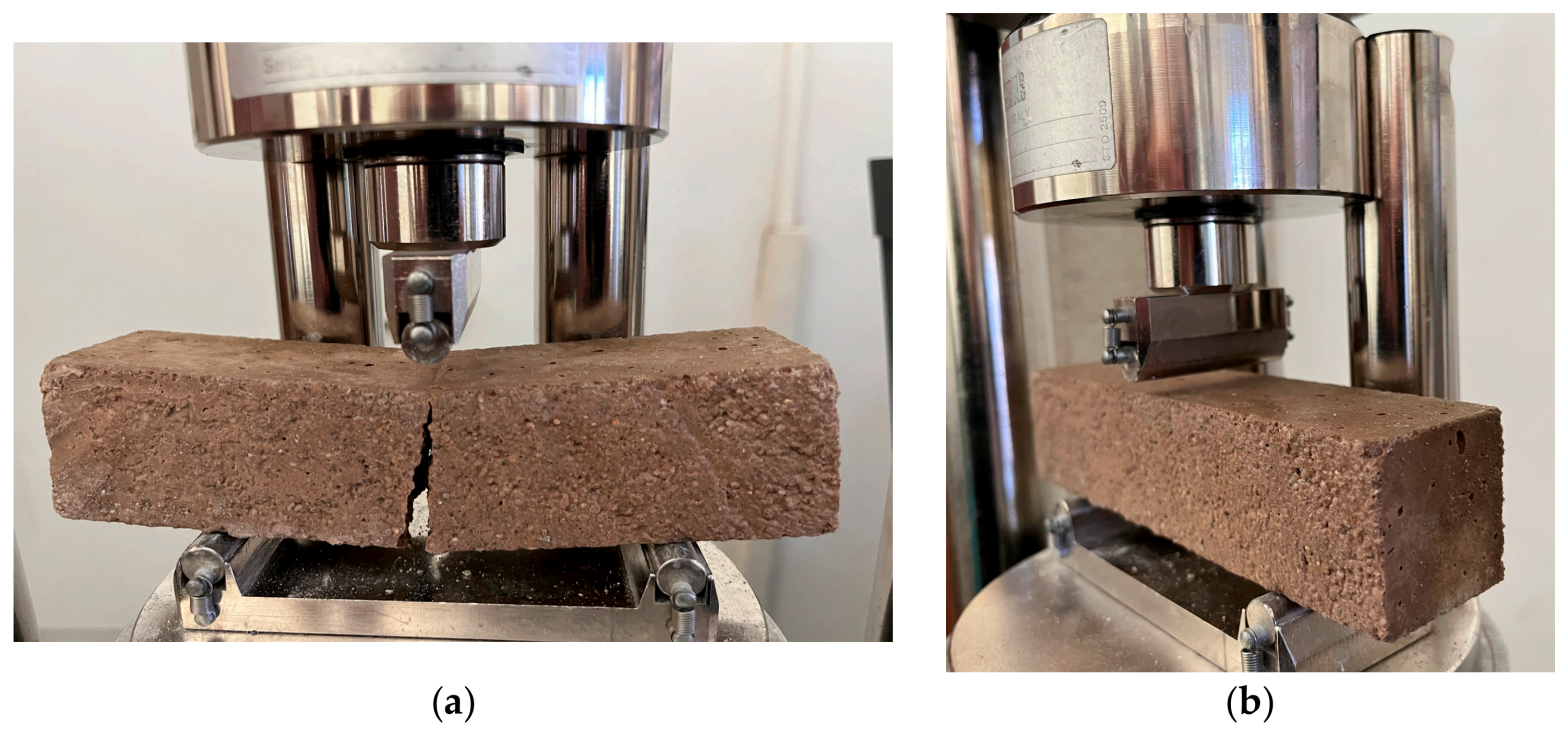
Testing flexural strength of mortar with red brick fine: (**a**) specimen after destruction; (**b**) three-point bending test scheme.

The flexural strength of all mortars was tested on the sets of three standard prism specimens of size 40 mm × 40 mm × 160 mm in the three-point bending test (Figure 9a), and the compressive strength was tested on the halves of the prisms remaining after the bending test (Figure 9b), using a Controls MC66 hydraulic press (CONTROLS S.p.A., Milan, Italy). Bulk density under the air-dry conditions was calculated for the same specimens prior to mechanical destructive testing, based on their mass and measured dimensions.

## 6. Results and Discussion

### 6.1. Consistency of the Fresh Mix

Due to the nature of the flow table test, which is not based solely on the friction of the mortar against the penetrating indenter (as in tests of pastes consistency carried out in the Vicat apparatus [87] or tests of mortars consistency tested in Novikov apparatus [88]), this test is considered an indicator of the workability/plastic workability of a material, which is linked to the rheological parameters of the mortar [89]. The flow table test measures how much a fresh mortar sample flows under the influence of table movements (shakes), which is directly related to the plastic deformation and workability of the mortar. In the literature, this type of test is often used as a reference for plastic/rheological parameters such as yield stress and plastic viscosity [90]. The results of the test, namely, the flow diameter determined for all the tested mortars, are summarized in Table 10; the presented values are the mean of two tests, each of which includes two measurements in perpendicular directions, thus four measurements altogether.

The effect of substituting cement with recycled CDW fines had a visible impact on the consistency/plasticity of the mortars. Taking into consideration that the fine derived from yellow “historic” brick was much finer (mode of 14.2 µm), not only from other recycled materials (modes in the range between 36.6 and 54.9 µm), but even from the Portland cement (mode of 36.6 µm), which resulted in a much more developed specific surface area (of 25.7k cm^2^/cm^3^), and therefore, a higher water demand, one could expect that this would be manifested also by a denser consistency. This expectation was confirmed—the flow diameter determined for the mortar with this fine was smaller by 29.4% compared to the flow diameter of the reference cement mortar. In contrast, the fines of grading curves similar to the cement’s curve (see Figure 5a) showed smaller effect on the consistency/plasticity of the fresh mix. The smallest impact was observed for red ceramic brick fine—flow diameter 18% smaller than the reference diameter of the pure cement mortar. This also was expected, because this red brick fine has the most similar grading (identical modes of size distributions of 36.6 µm) and closest specific surface area to Portland cement (11.7k cm^2^/cm^3^ and 11.5k cm^2^/cm^3^, respectively). The reduction in flow diameter registered for mixed CDW fine, AAC units fine, and ceramic block fine was as follows: 24%, 27% and 29%, respectively. Good plasticity of the mortar with mixed CDW fines can be explained by the fact that the mix contained also some small amount of cement–lime mortar fines.

### 6.2. Density and Mechanical Strength

**Figure 10:**
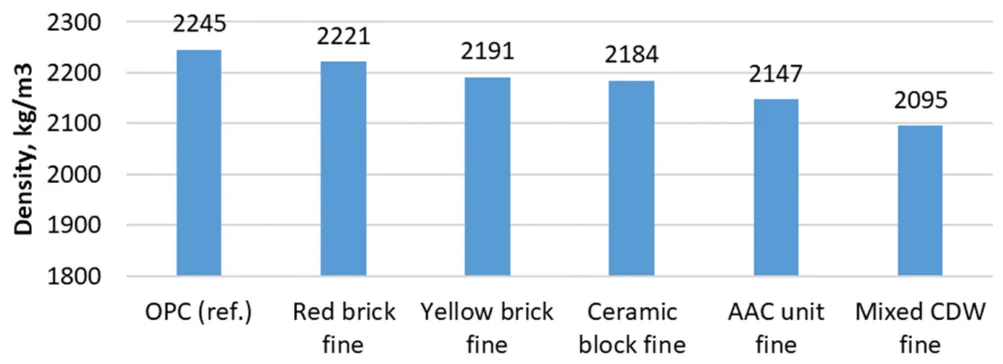
The apparent density of tested CDW fines after 28 days of water-curing (OPC—ordinary Portland cement).

**Figure 11:**
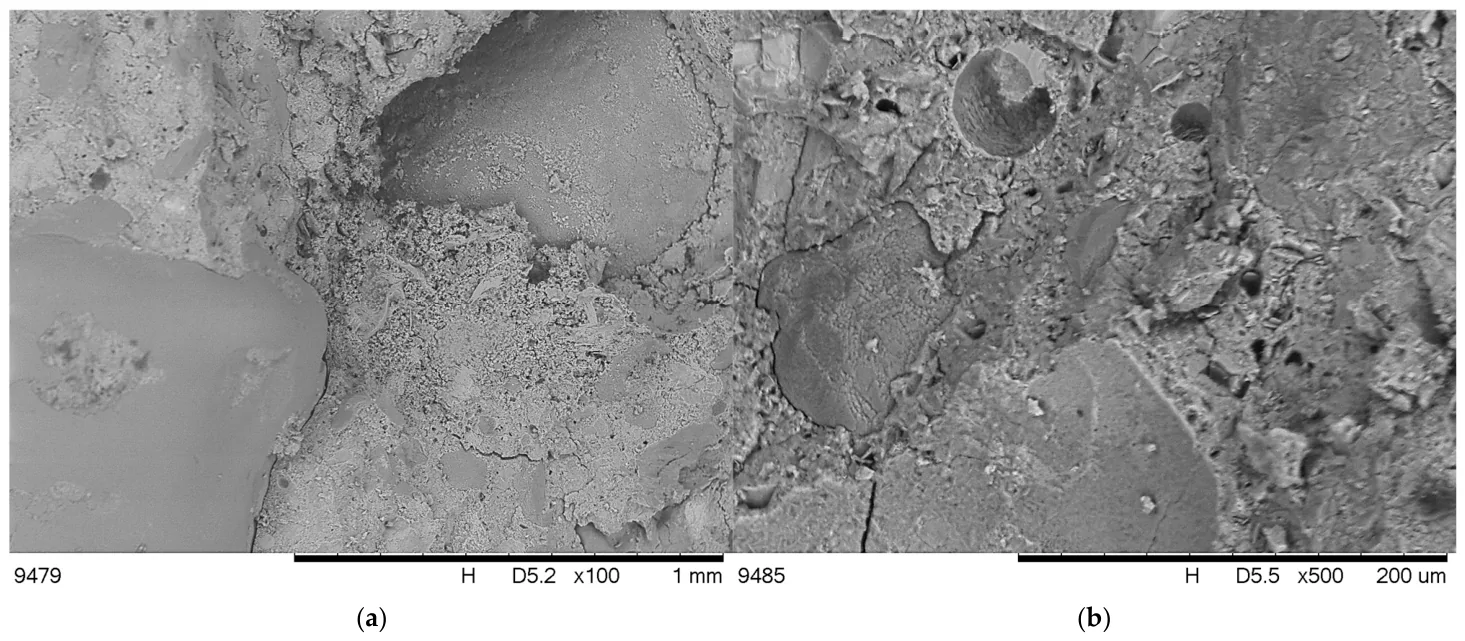
SEM micrographs (magnification: 1.0k×) of fractures of tested mortars with AAC units fines: (**a**) agglomeration of the fines at the surface of the grain of quartz sand; (**b**) increased porosity of the blended cementitious matrix.

The results of testing the bulk density of mortars with CDW fines after 28 days are shown in Figure 10. The data on the chart show only mean values, because coefficient of variation (CV) adopted very low values (between 0.5 and 2.0%) and the standard deviation (SD) or scatter of the results in the form of error bars would not be legible on the chart. This, however, confirms that it was possible to obtain very homogeneous solid materials of repeatable properties. The additional SEM microscopic observation of the mortars microstructure, helpful for analyzing the influence of are CDW fines on the properties are shown in Figure 11.

The density of the mortars with CDW fines adopted various values from the range between 2095 and 2221 kg/m^3^, thus lower than density of the reference standard (pure cement) mortar of 2245 kg/m^3^. This was partially due to the fact that the used recycled fines were characterized by a lower specific gravity (between 2332 and 2734 kg/m^3^) than Portland cement (3081 kg/m^3^), which during hydration additionally absorbs water and finally increases its mass by about 20% [55]. Moreover, SEM observations of fractures of hardened mortars containing recycled CDW fines, in the case of mortars with AAC fines and mixed CDW fines revealed agglomeration of finest particles in the immediate vicinity of the quartz sand aggregate grains (Figure 11a), as well as increased porosity of their blended cementitious matrices (Figure 11b), which influenced the microstructure of the composites and therefore lower their apparent density.

**Figure 12:**
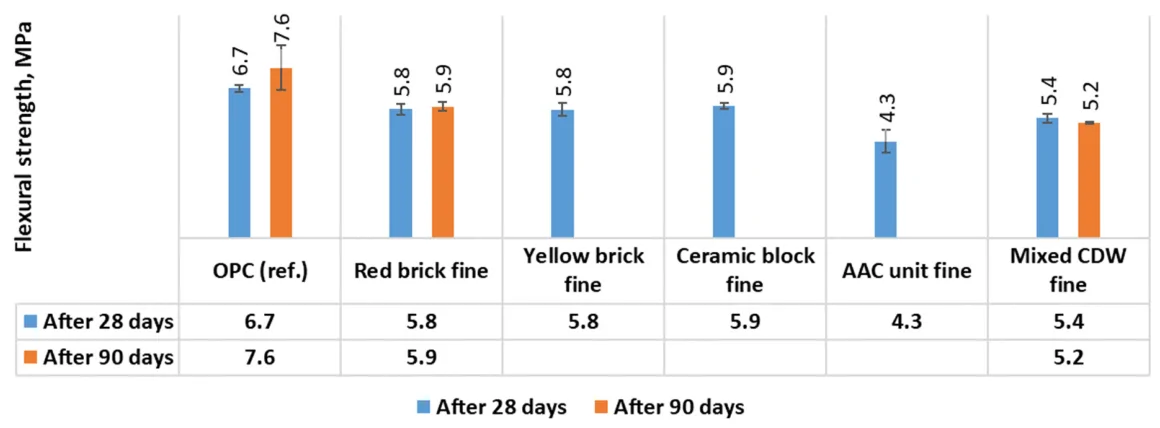
The flexural strength of tested mortars with CDW fines after 28/90 days of water-curing (OPC—ordinary Portland cement).

**Figure 13:**
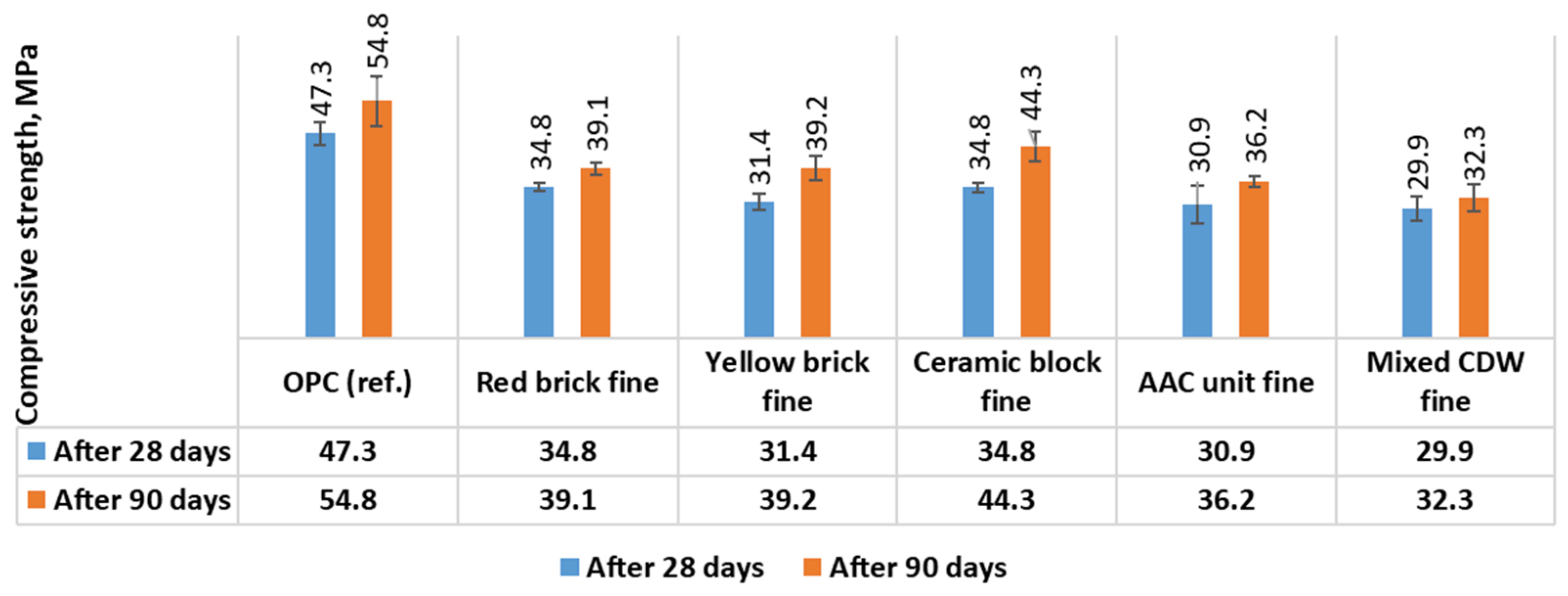
The compressive strength of tested mortars with CDW fines after 28/90 days of water-curing (OPC—ordinary Portland cement).

The above-mentioned SEM findings can be also an explanation of the strongest decrease in the mechanical strength of mortars with AAC unit fine and mixed CDW fine, compared to the properties of reference pure cement mortar. The results of testing the flexural strength and compressive strength of mortars with CDW fines after 28/90 days are shown in Figure 12 and Figure 13, respectively.

The results clearly indicate that ceramic fines performed better as SCMs than AAC-derived and mixed CDW fines—both when assessing the mortars for flexural strength (tensile strength when bending) and compressive strength. After 28 days of curing mortars containing ceramic fines achieved flexural strength of 5.8–5.9 MPa and compressive strength of 34.8 MPa (both red ceramics) and 31.4 MPa (yellow ceramics). Mortars containing AAC fine and mixed CDW fine after 28 days achieved significantly lower results: flexural strength of 4.3 MPa and 5.4 MPa, respectively, and compressive strength of 30 MPa on average. Additional flexural strength determinations performed after 90 days of curing showed that—unlike cement, which showed a 13.4% increase in strength—there was no additional increase in flexural strength in the case of the mortars with the analyzed CDW fines. In each case, however, the increase in compressive strength in further time was recorded. A relative increase in the 90-day strength to the 28-day strength was as following: 12.4% for mortars with red brick fine, 24.8% for mortars with yellow brick fine, 27.3% for mortars with ceramic block fine, 17.2% for mortars with AAC fine, and 8.0% for mortars with mixed CDW fine. In the case of pure cement reference mortar this increase was 19.9%.

An interesting observation was that after a longer curing period, the effect of both brick fines was ultimately the same: 39 MPa, while the other example of red ceramics, i.e., fines derived from ceiling blocks gave the best result of 90-day strength of 44.3 MPa. This last result is 23.7% lower that the strength of the 90-day-old pure cement reference mortar, yet higher that the designed class of applied Portland cement, i.e., 42.5 MPa. Thus, in the longer time, it was possible to obtain the theoretical desired 28-day strength of the composite.

Statistical analysis of the strength test results (especially the compressive strength test conducted on halves of prism specimens) again indicates that the authors were able to obtain materials with repeatable properties. Considering the coefficient of variation (see the bars on the charts in Figure 12 and Figure 13), the values are similar, and in most cases even smaller, than those of commercial cement. This indicates good repeatability of the tested materials and provides a basis for future structural investigations.

## 7. Summary

The presented research clearly confirms the necessity of performing a detailed characterization of construction and demolition waste (CDW) materials from renovation of old buildings—both in terms of the parent materials and fines derived from them, as it allows for a preliminary assessment of their potential as SCMs.

The study results indicate that materials containing hardened cement paste, i.e., fines obtained from recycled lightweight concrete (AAC) blocks, or a mix of various CDW containing these blocks, as well as fragments of cement–lime mortar, despite the expected content of unhydrated cement particles, did not exhibit particularly high residual alkalinity or high pozzolanic activity. This directly translated into poor results for the mechanical properties of mortars containing such fines. Significantly better results were obtained for fines derived from recycled clay ceramics elements. However, attention should be paid to the type/color of the recycled ceramic material, which indirectly results from the age of the ceramic product, as well as to its granulometric characteristics. Grinding different types of ceramics with the same grinding parameters yielded different results. The yellow “historical” brick was significantly finer, which resulted in a more developed specific surface area and, therefore, a higher water demand, while red brick and ceramic ceiling blocks, after grinding, were very similar to the Portland cement used in the research. This affected the rheology of the tested mortar mixes, their microstructure after hardening, and consequently, their mechanical properties determined after 28 days and 90 days of curing.

Considering the results of the completed research program, it was concluded that substituting Portland cement with various types of fine recycled CDW at a level of 25 wt%, which corresponded to a substitution of Portland clinker at a level of 33–36 wt%, produced very satisfactory results—especially in the context of recycled red ceramics (including bricks and ceiling blocks). The obtained results complement the current state of knowledge, as most studies described in the literature concern mortars in which cement substitution was performed at levels of 10%, 20%, and 30 wt%, with the latter level of substitution often cited by researchers as overestimated. Therefore, the 25 wt% substitution results complement those studies. These results also seem to be important, because the current standards do not recommend the use of recycled ceramic fines in cements in significant quantities, preferring recycled concrete fines. However, increasing the use of recycled ceramic fines in cement and concrete technology seems justified not only from an ecological, but also from a technical point of view, as demonstrated in this study.

The authors plan to continue this path of research—in subsequent stages they would like to focus on blends of recycled concrete fines and recycled ceramic fines in order to determine the optimal proportion of both recycled constituents with respect to concrete performance and durability.

## Figures and Tables

**Table 1:** Compositions of tested mortars: REF—reference standard mortar ^1^, M-AACF—mortar with recycled concrete fine from AAC units, M-RBF—mortar with recycled red brick fine, M-YBF—mortar with recycled yellow brick fine, M-CBF—mortar with recycled ceramic block fine, M-MF—mortar with recycled mixed fine.

Code	Mineral Binder [g]						Water [g]	Sand [g]
	OPC	Red Brick Fine	Yellow Brick Fine	Ceram. Block Fine	AAC Fine	Mixed Fine		
REF ^1^	450.0	0.0	0.0	0.0	0.0	0.0	225.0	1350.0
M-RBF	337.5	112.5	0.0	0.0	0.0	0.0	225.0	1350.0
M-YBF	337.5	0.0	112.5	0.0	0.0	0.0	225.0	1350.0
M-CBF	337.5	0.0	0.0	112.5	0.0	0.0	225.0	1350.0
M-AACF	337.5	0.0	0.0	0.0	112.5	0.0	225.0	1350.0
M-MF	337.5	0.0	0.0	0.0	0.0	112.5	225.0	1350.0

^1^ Composition of standard mortar acc. to EN-196-1 standard.

**Table 2:** Size distribution of CEN standard sand according to EN 196-1.

Square Mesh Size, mm	2.00	1.60	1.00	0.50	0.16	0.08
Total residue on the sieve, wt%	0	7 ± 5	33 ± 5	57 ± 5	87 ± 5	99 ± 1

**Table 3:** Selected characteristics of the used cement CEM I 42.5R based on the manufacturer’s declaration data (Cementownia “Odra” S.A., Opole, Poland).

Properties	Characteristic	Information/Value
Chemical Characteristics, wt%	Ignition loss	2.5
	Sulphate ions content SO_3_^−^	2.5
	Chloride ions content Cl^−^	0.03
Physical properties	Beginning of setting time [min]	200
	Stability of volume [mm]	1.0
	Hydration heat (after 41 h), J/g	325

**Table 4:** Statistical parameters describing the particle size distribution and specific surface area of Portland cement CEM I 42.5R used in the study.

Statistical Parameter of PSD	Value
D_min_ [µm]	0.2
D_10_ [µm]	7.1
D_50_ (median) [µm]	30.5
D_m_ (mean) [µm]	37.2
D_90_ [µm]	76.0
Mode [µm]	36.6
CV	78.7
SPA ^1^ [cm^2^/cm^3^]	15,068

^1^ Calculated from the PSD, assuming the spherical shape of the particles.

**Table 5:** Experimentally determined characteristics of ceramic CDW that were source materials for recycled fines used in the study: SD—standard deviation; CV—coefficient of variation.

Property	Red Brick			Yellow Brick			Ceramic Blocks		
	Mean	SD	CV [%]	Mean	SD	CV [%]	Mean	SD	CV [%]
Bulk density, kg/m^3^	1649	112	6.9	1559	94	6.1	1546	114	7.4
Specific gravity, kg/m^3^	2579	3	0.1	2332	12	0.5	2734	20.0	0.7
Water absorption (by wt%)	20.8	1.0	4.9	37.7	1.7	5.3	19.5	0.9	4.6
Water absorption (by vol%)	34.3	3.0	8.9	50.9	3.1	6.1	30.2	2.6	8.5
Porosity ^1^ [%]	36.1			33.2			43.4		
Tightness ^1^ [-]	0.64			0.67			0.57		

^1^ Calculated based on the specific gravity and bulk density (of specimen dried to constant mass).

**Table 6:** Experimentally determined characteristics of cementitious CDW that were source materials for recycled fines used in the study: SD—standard deviation; CV—coefficient of variation.

Property	AAC Units			Cement–Lime Mortar		
	Mean	SD	CV [%]	Mean	SD	CV [%]
Bulk density, kg/m^3^	464	52.0	11.2	1719	125	7.3
Specific gravity, kg/m^3^	2478	9.0	0.4	2579	7.0	0.3
Water absorption (by wt%)	64.1	5.0	7.8	12.7	0.7	5.4
Water absorption (by vol%)	29.8	4.2	14.2	21.9	2.2	10.3
Porosity ^1^ [%]	81.2			33.3		
Tightness ^1^ [-]	0.19			0.67		

^1^ Calculated based on the specific gravity and bulk density (of specimen dried to constant mass).

**Table 7:** Averaged L, a, b coordinates, ∆*E*^∗^_*ab*_ and representations of colors converted to the sRGB and CMYK scale determined for the crushed ceramic fragments.

Property	Yellow Brick			Ceramic Blocks			Red Brick		
	Mean	SD	CV [%]	Mean	SD	CV [%]	Mean	SD	CV [%]
Results of the examination of ceramic fragments									
L	65.31	1.02	1.56	52.46	1.95	3.71	51.42	4.88	9.50
a	8.80	0.76	8.62	17.61	0.64	3.64	19.61	2.40	12.22
b	20.60	0.46	2.25	25.06	0.73	2.93	28.82	4.01	13.93
∆*E*^∗^_*ab*_ ^1^	2.20			2.20			7.80		
sRGB	184 152 122			163 113 83			164 109 74		
CMYK	29% 38% 54% 2%			31% 56% 70% 12%			30% 58% 76% 13%		
Results of the examination of ceramic fines									
L	70.25	0.29	0.41	54.51	0.08	0.15	53.30	0.47	0.88
a	8.19	0.06	0.77	16.87	0.07	0.42	22.59	0.09	0.41
b	20.89	0.12	0.56	24.79	0.04	0.15	33.82	0.88	2.59
∆*E*^∗^_*ab*_ ^1^	0.27			0.19			0.74		
sRGB	197 166 134			168 119 88			174 111 70		
CMYK	24% 33% 49% 0%			30% 53% 68% 10%			26% 59% 80% 10%		

^1^ Mean value out of 5 results of ∆*E*^∗^_*ab*_ calculated towards the first result selected as the representative.

**Table 8:** Statistical parameters describing the particle size distribution and specific surface area of tested CDW fines before and after mechanical activation.

Statistical Parameter of PSD	Red Brick Fine	Yellow Brick Fine	Ceram. Block Fine	AAC Unit Fine	Cement–Lime Plaster Fine	Mixed CDW Fine
D_min_ [µm]	0.2	0.2	0.2	2.9	2.0	0.2
D_10_ [µm]	8.5	0.7	6.9	10.8	9.5	10.3
D_50_ (median) [µm]	30.7	14.8	36.7	33.0	47.3	39.8
D_m_ (mean) [µm]	37.1	22.0	43.9	38.6	59.6	46.4
D_90_ [µm]	74.1	51.3	90.9	73.1	128.9	91.4
Mode [µm]	36.6	14.2	54.9	36.8	72.3	48.1
CV	75.8	106.6	78.7	68.9	81.9	72.0
SPA ^1^ [cm^2^/cm^3^]	11,749	25,761	10,247	2700	2656	8144

^1^ Calculated from the PSD, assuming the spherical shape of the particles.

**Table 9:** Temperature and pH of aqueous solution washed out from Portland cement CEMI 42.5R and ground CDW fines determined following the ISO 1039 procedure.

Parameter	OPC CEMI 42.5R	Red Brick Fine	Yellow Brick Fine	Ceramic Block Fine	AAC Unit Fine	Mixed CDW Fine
pH	12.92	9.72	8.24	10.13	8.29	9.06
Temperature [°C]	19.2	18.7	18.1	18.0	20.0	19.7

**Table 10:** Flow table tests results performed for pure Portland cement-based reference mortar and for mortars containing 25% of CDW materials in binder blends.

Parameter	REF	M-RBF	M-YBF	M-CBF	M-AACF	M-MF
Flow diameter, mm	197	162	139	141	144	149
SD, mm	1	2	1	1	2	1
CV, %	0.7	1.2	0.7	0.6	1.2	0.7

## Data Availability

The original contributions presented in this study are included in the article. Further inquiries can be directed to the corresponding author.

## Abbreviations

The following abbreviations are used in this manuscript:

CDW	Construction and demolition waste (also known as “C&DW” or “C&D waste”)
AAC	Autoclaved aerated concrete
SCM	Supplementary cementing material
OPC	Ordinary Portland cement
CEM	Cement
EN	European Norm (Standard)
F	Recycled concrete fines
C-S-H	Calcium silicate hydrate (also known as CSH)
AFm	Monosulfate
AFt	Ettringite
C-F-H	Calcium ferrite hydrate (also known as CFH)
PSD	Particle size distribution
NDT	Non-destructive testing
HPC	High-performance concrete
C-A-S-H	Calcium aluminum silicate hydrate (also known as CASH or C(A)SH)
N-A-S-H	Sodium aluminum silicate hydrate (also known as NASH)
CEN	Standardized sand
b/a	Binder-to-aggregate ratio
w/c	Water-to-cement ratio
REF	Reference standard mortar
M-RBF	Mortar with recycled red brick fine
M-YBF	Mortar with recycled yellow brick fine
M-CBF	Mortar with recycled ceramic block fine
M-AACF	Mortar with recycled concrete fine from AAC units
M-MF	Mortar with recycled mixed fine
D_min_	Minimal diameter
D_10_	Diameter/size below which 10% of all particles are found
D_50_	Median diameter
D_m_	Mean diameter
D_90_	Diameter/size below which 90% of all particles are found
CV	Coefficient of variation
SPA	Specific (surface) area
SD	Standard deviation
pH	Concentration of hydrogen ions (H^+^) in solution
L	Coordinate in the CIELAB space corresponding to lightness
a	Coordinate in the CIELAB space corresponding to red and green
b	Coordinate in the CIELAB space corresponding to blue and yellow
CIELAB	International Commission on Illumination color space (also known as L*a*b*)
∆*E*^∗^_*a**b*_	Distance between two points in the colorimetric CIELAB space
sRGB	“Standardized Red, Green, Blue” color space
CMYK	“Cyan, Magenta, Yellow, Black” color model
LC	Lightweight concrete
LST	Light scattering theory
CAS	Chemical abstracts service
SEM	Scanning electron microscope

## References

[1] USGS Mineral Commodity Summaries 2025. https://pubs.usgs.gov/periodicals/mcs2025/mcs2025_ver.1.0.pdf.

[2] Cheng D., Reiner D.M., Yang F., Cui C., Meng J., Shan Y., Liu Y., Tao S., Guan D. (2023). Projecting Future Carbon Emissions from Cement Production in Developing Countries. Nat. Commun..

[3] Chen K., Wang J., Yu B., Wu H., Zhang J. (2021). Critical Evaluation of Construction and Demolition Waste and Associated Environmental Impacts: A Scientometric Analysis. J. Clean. Prod..

[4] Wu H., Zuo J., Zillante G., Wang J., Yuan H. (2019). Status Quo and Future Directions of Construction and Demolition Waste Research: A Critical Review. J. Clean. Prod..

[5] Chandrappa R., Das D.B., Chandrappa R., Das D.B. (2024). Construction and Demolition Waste. Solid Waste Management: Principles and Practice.

[6] UN Secretary-General’s Advisory Board on Zero Waste Constructing Sustainable Infrastructure Towards a Circular Economy. https://unhabitat.org/sites/default/files/2024/10/construction_waste.pdf.

[7] Han T., Aponte D., Valls S., Bizinotto M.B. (2025). Impact of Recycled Concrete and Ceramic Fillers on the Performance of Cementitious Systems: Microstructural, Mechanical, and Durability Aspects. Recycling.

[8] Ragossnig A.M. (2020). Construction and Demolition Waste—Major Challenges Ahead!. Waste Manag. Res..

[9] Menegakis M., Damigos D. (2018). A Review on Current Situation and Challenges of Construction and Demolition Waste Management. Curr. Opin. Green Sustain. Chem..

[10] Eurostat Waste Statistics (Data Extracted in September 2024). https://ec.europa.eu/eurostat/statistics-explained/index.php?title=Waste_statistics.

[11] Joint Research Centre (European Commission) (2022). Background Data Collection and Life Cycle Assessment for Construction and Demolition Waste (CDW) Management.

[12] Directorate-General for Internal Market, Industry, Entrepreneurship and SMEs (European Commission) (2018). Development and Implementation of Initiatives Fostering Investment and Innovation in Construction and Demolition Waste Recycling Infrastructure.

[13] Hafez H., Kurda R., Kurda R., Al-Hadad B., Mustafa R., Ali B. (2020). A Critical Review on the Influence of Fine Recycled Aggregates on Technical Performance, Environmental Impact and Cost of Concrete. Appl. Sci..

[14] Łukowski P., Horszczaruk E., Seul C., Strzałkowski J. (2022). Mechanical Strength and Thermal Properties of Cement Concrete Containing Waste Materials as Substitutes for Fine Aggregate. Materials.

[15] Yuan X., Tian Y., Ahmad W., Ahmad A., Usanova K.I., Mohamed A.M., Khallaf R. (2022). Machine Learning Prediction Models to Evaluate the Strength of Recycled Aggregate Concrete. Materials.

[16] Li C., Zhang X., Cao X., Yu Y. (2025). Sustainable Performance and Interfacial Characteristics of Fully Recycled Concrete with Combined Recycled Concrete, Brick, and Ceramic Aggregates. Sustainability.

[17] Debieb F., Kenai S. (2008). The use of coarse and fine crushed bricks as aggregate in concrete. Constr. Build. Mater..

[18] Yunusa M., Zhang X., Cui P., Tian X. (2022). Durability of Recycled Concrete Aggregates Prepared with Mechanochemical and Thermal Treatment. Materials.

[19] Oliveira D.R.B., Leite G., Possan E., Filho J.M. (2023). Concrete powder waste as a substitution for Portland cement for environment-friendly cement production. Constr. Build. Mater..

[20] Rodriguez-Morales J., Burciaga-Diaz O., Gomez-Zamorano L.Y., Escalante-Garcia J.I. (2024). Transforming Construction and Demolition Waste Concrete as a Precursor in Sustainable Cementitious Materials: An Innovative Recycling Approach. Resour. Conserv. Recycl..

[21] De Brabandere L., Grigorjev V., Van den Heede P., Nachtergaele H., Degezelle K., De Belie N. (2025). Using Fines from Recycled High-Quality Concrete as a Substitute for Cement. Sustainability.

[22] Li J., Deng X., Lu Z., Li X., Hou L., Jiang J., Yang F., Zhang J., He K. (2024). Recycled concrete fines as a supplementary cementitious material: Mechanical performances, hydration, and microstructures in cementitious systems. Case Stud. Constr. Mater..

[23] Tokareva A., Kaassamani S., Waldmann D. (2023). Fine demolition wastes as Supplementary cementitious materials for CO_2_ reduced cement production. Constr. Build. Mater..

[24] Mohammed S. (2017). Processing, effect and reactivity assessment of artificial pozzolans obtained from clays and clay wastes: A Review. Constr. Build. Mater..

[25] Wu H., Liang C., Wang C., Ma Z. (2022). Properties of green mortar blended with waste concrete-brick powder at various components, replacement ratios and particle sizes. Constr. Build. Mater..

[26] Li S., Chen G., Xu Z., Luo X., Gao J. (2022). Particle-size effect of recycled clay brick powder on the pore structure of blended cement paste. Constr. Build. Mater..

[27] Zheng L., Ge Z., Yao Z., Gao Z. Mechanical Properties of Mortar with Recycled Clay-Brick-Powder. Proceedings of the ICCTP 2011: Towards Sustainable Transportation Systems.

[28] Shao J., Gao J., Zhao Y., Chen X. (2019). Study on the pozzolanic reaction of clay brick powder in blended cement pastes. Constr. Build. Mater..

[29] Lam N.T., Nguyen D.T., Nguyen D.L. (2021). Potential use of clay brick waste powder and ceramic waste aggregate in mortar. Constr. Build. Mater..

[30] Arif R., Khitab A., Kırgız M.S., Khan R.B.N., Tayyab S., Khan R.A., Anwar W., Arshad M.T. (2021). Experimental analysis on partial replacement of cement with brick powder in concrete. Case Stud. Constr. Mater..

[31] Sokołowska J.J., Załęgowski K. (2018). Ultrasonic quality assessment of polymer-cement concrete with pet waste as the aggregate. Arch. Civ. Eng..

[32] Tawfik T.A., Sičáková A., Kuzielová E., Kušnír Š., Eštoková A., Bálintová M., Junáková N. (2024). Sustainable reuse of waste ceramic tiles powder and waste brick powder as a replacement for cement on green high strength concrete properties. Innov. Infrastruct. Solut..

[33] Andrade R.M., Araújo A.J.M., Raimundo R.A., Dutra R.P.S., Campos L.F.A., Vivacqua C.A., Pinho A.L.S., Macedo D.A. (2024). Understanding the Processing of Mullite-Based Ceramics from Kaolin Waste: Structural and Phase Characterization. J. Aust. Ceram. Soc..

[34] Liang G., Luo L., Yao W. (2022). Reusing waste red brick powder as partial mineral precursor in eco-friendly binders: Reaction kinetics, microstructure and life-cycle assessment. Resour. Conserv. Recycl..

[35] Sharmin S., Sarker P.K., Biswas W.K., Abousnina R.M., UJaved U. (2024). Characterization of waste clay brick powder and its effect on the mechanical properties and microstructure of geopolymer mortar. Constr. Build. Mater..

[36] Pachta V., Konopisi S., Stefanidou S. (2012). The influence of brick dust and crushed brick on the properties of lime-based mortars exposed at elevated temperatures. Constr. Build. Mater..

[37] Shah M.U., Usman M., Hanif M.U., Naseem I., Farooq S. (2021). Utilization of Solid Waste from Brick Industry and Hydrated Lime in Self-Compacting Cement Pastes. Materials.

[38] De Matos P.R., Sakata R.D., Onghero L., Uliano V.G., de Brito J., Campos C.E.M., Gleize P.J.P. (2021). Utilization of ceramic tile demolition waste as supplementary cementitious material: An early-age investigation. J. Build. Eng..

[39] El-Dieb A.S., Kanaan D.M. (2018). Ceramic waste powder an alternative cement replacement—Characterization and evaluation. Sustain. Mater. Technol..

[40] Tanash A.O., Muthusamy K., Budiea A.M.A., Fauzi M.A., Jokhio G., Jose R. (2023). A review on the utilization of ceramic tile waste as cement and aggregates replacement in cement based composite and a bibliometric assessment. Clean. Eng. Technol..

[41] Straits Research Autoclaved Aerated Concrete (AAC) Market Size & Outlook. https://straitsresearch.com/report/autoclaved-aerated-concrete-market.

[42] Shrestha A.R., Xia J., Di Sarno L., Chin C.S. (2024). Feasibility study of utilizing Autoclaved Aerated Concrete (AAC) waste for the production of cold bonded lightweight artificial aggregate using high volume fly ash (HVFA) binders. Constr. Build. Mater..

[43] Volk R., Steins J.J., Stemmermann P., Schultmann F. (2022). Comparison of different post-demolition autoclaved aerated concrete (AAC) recycling options. IOP Conf. Ser. Earth Environ. Sci..

[44] Radina L., Sprince A., Pakrastins L., Gailitis R., Sakale G. (2023). Potential Use of Construction Waste for the Production of Geopolymers: A Review. Mater. Proc..

[45] Lam N.N. (2021). Recycling of AAC waste in the manufacture of autoclaved aerated concrete in Vietnam. Int. J. GEOMATE.

[46] Sun D., Huang N., Liu K., Tang J., Rong N., Wang A., Guan Y., Liang P., Deng Y. (2023). Effect of recycled fine powder on autoclaved aerated concrete: Gas-foaming, physic-mechanical property and hydration products. J. Build. Eng..

[47] Wang S., Zhang G., Gunasekara C., Law D., Tan Y., Sun W. (2025). A Scientific Review of Recycling Practices and Challenges for Autoclaved Aerated Concrete in Sustainable Construction. Buildings.

[48] Łukowski P., Salih A., Sokołowska J.J. (2018). Frost resistance of concretes containing ground granulated blast-furnace slag. MATEC Web Conf..

[49] Sokołowska J.J. (2025). Utilizing Sakurajima Volcanic Ash as a Sustainable Partial Replacement for Portland Cement in Cementitious Mortars. Sustainability.

[50] Sokołowska J.J., Chmielewska B., Czarnecki L., Garbacz A., Wang R., Frigione M., Aguiar J.B. (2025). Carbon Footprint and CO_2_ Emissions in the Concrete-Polymer Composites Technology. Concrete-Polymer Composites in Circular Economy, Proceedings of the ICPIC 2023, Warsaw, Poland, 16–19 September 2023.

[51] (2023). Cement—Part 6: Cement with Recycled Building Materials.

[52] (2008). Aggregates for Concrete.

[53] (2022). Tests for Geometrical Properties of Aggregates—Part 1: Determination of Particle Size Distribution—Sieving Method.

[54] Czarnecki L., Broniewski T., Hening O. (2006). Chemia w Budownictwie.

[55] Kurdowski W. (2014). Cement and Concrete Chemistry.

[56] (2016). Methods of Testing Cement—Part 1: Determination of Strength.

[57] (2011). Cement—Part 1: Composition, Specifications and Conformity Criteria for Common Cements.

[58] (2012). Fly Ash for Concrete—Part 1: Definition, Specifications and Conformity Criteria.

[59] (2002). Mixing Water for Concrete-Specification for Sampling, Testing and Assessing the Suitability of Water, Including Water Recovered from Processes in the Concrete Industry, as Mixing Water for Concrete.

[60] (2006). Natural Stone Test Methods—Determination of Real Density and Apparent Density, and of Total and Open Porosity.

[61] Kreimeyer R. (1987). Some Notes on the Firing Colour of Clay Bricks. Appl. Clay Sci..

[62] Stępkowska E.T., Jefferis S.A. (1992). Influence of Microstructure on Firing Colour of Clays. Appl. Clay Sci..

[63] Rathossi C., Pontikes Y. (2010). Effect of Firing Temperature and Atmosphere on Ceramics Made of NW Peloponnese Clay Sediments. Part I: Reaction Paths, Crystalline Phases, Microstructure and Colour. J. Eur. Ceram. Soc..

[64] Mastrotheodoros G.P., Beltsios K.G. (2022). Pigments—Iron-Based Red, Yellow, and Brown Ochres. Archaeol. Anthropol. Sci..

[65] Dizhur D., Lumantarna R., Biggs D.T., Ingham J.M. (2017). In-Situ Assessment of the Physical and Mechanical Properties of Vintage Solid Clay Bricks. Mater. Struct..

[66] Nix Color Sensor Converter. https://www.nixsensor.com/free-color-converter/.

[67] Sharma G., Wu W., Dalal E.N. (2005). The CIEDE2000 Color-Difference Formula: Implementation Notes, Supplementary Test Data, and Mathematical Observations. Color Res. Appl..

[68] Zhang Z., Shang X., Li G., Wang G. (2023). Just Noticeable Difference Model for Images with Color Sensitivity. Sensors.

[69] Neville A.M. (2011). Properties of Concrete.

[70] (2016). Concrete—Specification, Performance, Production and Conformity.

[71] Pavlík Z., Pokorný J., Pavlíková M., Zemanová L., Záleská M., Vyšvařil M., Žižlavský T. (2019). Mortars with Crushed Lava Granulate for Repair of Damp Historical Buildings. Materials.

[72] (2004). Products and Systems for the Protection and Repair of Concrete Structures—Test Methods—Determination of Resistance to Carbonation.

[73] Morandeau A., Thiéry M., Dangla P. (2014). Investigation of the carbonation mechanism of CH and C-S-H in terms of kinetics, microstructure changes and moisture properties. Cem. Concr. Res..

[74] Woyciechowski P., Sokołowska J. (2017). Self-terminated carbonation model as an useful support for durable concrete structure designing. Struct. Eng. Mech..

[75] Miranda V., Riccio D., Ruello G., Lattanzi R. (2023). A Novel Electromagnetic Method to Interpret Scattering Suppression from Spheres. 2023 Seventeenth International Congress on Artificial Materials for Novel Wave Phenomena (Metamaterials).

[76] Bohren C.F., Huffman D.R. (2010). Absorption and Scattering of Light by Small Particles.

[77] Sokołowska J.J., Załegowski K., Czarnecki L. (2025). Measuring of the polymer concrete maturity using non-destructive ultrasonic method. Arch. Civ. Mech. Eng..

[78] (2017). Geotechnical Investigation and Testing—Identification and Classification of Soil. Part 1: Identification and Description.

[79] (2017). Standard Practice for Classification of Soils for Engineering Purposes (Unified Soil Classification System).

[80] (2021). Soil, Treated Biowaste and Sludge—Determination of pH.

[81] Rörig-Dalgaard I., Charola A.E. (2016). Influence of pH during chemical weathering of bricks: Long-term exposure. Materials, Systems and Structures in Civil Engineering 2016—Segment on Historical Masonry.

[82] El-Midany A.A., Mahmoud H.M. (2015). Mineralogical, physical and chemical characteristics of historic brick-made structures. Mineral. Petrol..

[83] Czarnecki L. (2004). Concrete According to the PN-EN 206-1 Standard—Commentary.

[84] Zieliński K.K. (2015). Fundamentals of Concrete Technology.

[85] Taylor H.F.W. (1997). Cement Chemistry.

[86] (2007). Methods of Test for Mortar for Masonry: Determination of Consistence of Fresh Mortar (by Flow Table).

[87] Lee J.-C., Yi C.-Y. (2022). Setting Process Monitoring of Cement Paste Using Electromechanical Impedance of Piezoelectric Patch. Materials.

[88] Jaworska B., Stańczak D., Tarańska J., Jaworski J. (2021). The Influence of Cement Substitution by Biomass Fly Ash on the Polymer–Cement Composites Properties. Materials.

[89] Senff L., Barbetta P.A., Repette W.L., Hotza D., Paiva H.M., Ferreira V.M., Labrincha J.A. (2009). Mortar composition defined according to rheometer and flow table tests using factorial designed experiments. Constr. Build. Mater..

[90] Shin T.Y., Kim J.H. (2021). First step in modeling the flow table test to characterize the rheology of normally vibrated concrete. Cem. Concr. Res..

